# The Stibium Bond or the Antimony-Centered Pnictogen Bond: The Covalently Bound Antimony Atom in Molecular Entities in Crystal Lattices as a Pnictogen Bond Donor

**DOI:** 10.3390/ijms23094674

**Published:** 2022-04-23

**Authors:** Arpita Varadwaj, Pradeep R. Varadwaj, Helder M. Marques, Koichi Yamashita

**Affiliations:** 1 Department of Chemical System Engineering, School of Engineering, The University of Tokyo, Tokyo 113-8656, Japan; varadwaj.arpita@gmail.com (A.V.); yamasita@chemsys.t.u-tokyo.ac.jp (K.Y.); 2 Molecular Sciences Institute, School of Chemistry, University of the Witwatersrand, Johannesburg 2050, South Africa; helder.marques@wits.ac.za

**Keywords:** pnictogen bonding, antimony as pnictogen bond donor, non-bonded geometry, directionality, crystal structure analysis, ICSD and CSD database analyses, MESP model description, sum of the van der Waals radii concept, pro-molecular charge-density based IGM-δ*g* analysis

## Abstract

A stibium bond, i.e., a non-covalent interaction formed by covalently or coordinately bound antimony, occurs in chemical systems when there is evidence of a net attractive interaction between the electrophilic region associated with an antimony atom and a nucleophile in another, or the same molecular entity. This is a pnictogen bond and are likely formed by the elements of the pnictogen family, Group 15, of the periodic table, and is an inter- or intra-molecular non-covalent interaction. This overview describes a set of illustrative crystal systems that were stabilized (at least partially) by means of stibium bonds, together with other non-covalent interactions (such as hydrogen bonds and halogen bonds), retrieved from either the Cambridge Structure Database (CSD) or the Inorganic Crystal Structure Database (ICSD). We demonstrate that these databases contain hundreds of crystal structures of various dimensions in which covalently or coordinately bound antimony atoms in molecular entities feature positive sites that productively interact with various Lewis bases containing O, N, F, Cl, Br, and I atoms in the same or different molecular entities, leading to the formation of stibium bonds, and hence, being partially responsible for the stability of the crystals. The geometric features, pro-molecular charge density isosurface topologies, and extrema of the molecular electrostatic potential model were collectively examined in some instances to illustrate the presence of Sb-centered pnictogen bonding in the representative crystal systems considered.

## 1. Introduction

Naming a chemical interaction formed by an element of the periodic table and its subsequent characterization readily enable one to identify its status and visualize what its role could be in the rational design of complex systems [1,2]. After identifying, in the last century, the possible existence of “close contacts” between atomic domains in molecules, molecular complexes and crystals, considerable efforts were made to classify, characterize, and name them [2]. One such close contact that occurs between atomic domains is widely known as the hydrogen bond; it is a non-covalent (chemical) interaction caused by a force of attraction between atomic sites of opposite polarity, following the fundamental law of electrostatics, i.e., Coulomb’s law, that states that opposite charges attract whereas like charges repel each other when in close proximity. As Arunan pointed out in an unpublished study [3], over fifty different proposals have been put forward to date on the characterization of hydrogen bonds, dating back to the time of Werner (1902) [4], Hantzsch (1910) [5] and Pfeifer (1913) [6], among many others [3]. However, a new definition of hydrogen bonds was adopted as recently as 2011 [7], listing many characteristics and indicators of such interactions that emerged from a variety of computational and experimental measurements, including IR, Raman, and NMR spectroscopic techniques, and X-ray and neutron diffraction measurements. 

First-principles [8,9,10,11,12] and density functional theory [13,14,15] methods have also been developed and applied to calculate the energy of hydrogen bonds in chemical systems and various spectroscopic and geometrical signatures, as well as the nature of the charge density profile associated with the surfaces of the interacting atomic domains in molecular entities responsible for these interactions. 

Following the definition and delineation of the characteristic features of hydrogen bonding, a similar definition, together with a set of characteristic features, was recommended for halogen bonding in 2013 [16]. This appears to have been prompted by the realization that thousands of solid-state systems were synthesized during the last decade that conceived directional intermolecular interactions driven by covalently bound elements of Group 17. There is very little difference between the definitions provided for hydrogen bonds and halogen bonds (except that the word “hydrogen” replaces “halogen” in the latter, of course); both atom types in molecular entities must feature electrophilic regions on their electrostatic surfaces and be capable of accepting some fractional charge density from an electron density donor fragment in the partner molecular species when in its close proximity.

Since the definition and characteristic features of a hydrogen bond were found to be transferable to the elements of Group 17 of the periodic table, they are, in principle, transferable to other elements, for instance, those of Groups 14–16, as well. In fact, this was pointed out in an article published in 2017 that recommended a definition for and described a number of characteristic features of chalcogen bonds in chemical systems (Group 16) [17]. 

We believe that the same definition is also transferable to the family of pnictogen bonds formed by the pnictogen elements, i.e., Group 15. 

*A pnictogen bond in chemical systems occurs when there is evidence of a net attractive interaction between the electrophilic region associated with a covalently or coordinately bound pnictogen atom in a molecular entity and a nucleophile in another, or the same molecular entity. It is formed by the elements of the pnictogen family, Group 15, of the periodic table, and is an inter- or intra-molecular non-covalent interaction.*

Clearly, when a covalently bound nitrogen atom in a molecular entity behaves as an electrophile toward a Lewis base, one would be tempted to call such an attractive engagement a nitrogen bond, in which, the nitrogen atom is a pnictogen bond donor. The same conceptual framework is transferable to covalently bound phosphorous, arsenic, antimony, bismuth and (in principle, anyway) moscovium when they display positive sites and have the ability to donate the electrophile to a Lewis base to form phosphorous, arsenic, stibium, bismuth, and moscovium bonds, respectively. 

This overview is focused on the possible occurrence of antimony-centered pnictogen bonds (or simply, *stibium bonds*) in chemical systems that have been known since the last century. In our view, it is an overlooked non-covalent interaction yet to be fully appreciated by, among others, computational and supramolecular chemists, gas-phase spectroscopists and materials scientists. This is not surprising given that the term “pnictogen bond” was coined only recently [18], and that the majority of studies of non-covalent interactions in the last and current centuries have been focused on arriving at a basic understanding of hydrogen bonding and van der Waals interactions. Legon has argued that pnictogen bonding (and other interactions) was recognized long before it was specifically named [19].

In the past and current decades, numerous studies have emerged on halogen [20,21,22,23] and chalcogen bonding [24,25,26,27,28,29,30], which are responsible for numerous crystal systems; in contrast, there have only been a small number of original research articles, reviews, and overviews that have focused on pnictogen bonding [19,31,32,33,34,35,36,37,38,39,40,41,42,43,44,45,46,47,48,49,50].

For the reasons given in the following sections, we believe that supramolecular pnictogen bonds are one of the cornerstones for the development of novel functional materials [51,52,53] and occur simultaneously with other non-covalent interactions (viz. halogen bonding, tetrel bonding, and hydrogen bonding), although their importance may only be fully appreciated in the years to come. In this overview, we provide several illustrative examples of crystal structures deposited in the Inorganic Crystal Structure Database (ICSD) [54,55]) and the Cambridge Structural Database (CSD) [56] in which the identification and characterization of stibium bonds were apparently overlooked, even though they play a fundamental role in the assembly of these solid-state materials. This study is therefore expected to assist researchers in the future design of novel functional materials featuring stibium bonds. With this in mind, we attempt to highlight, using illustrative examples, the occurrence and modes of interaction of such bonds.

## 2. Antimony in Molecular Entities, Materials Design and Discovery 

Antimony, the fourth member of the pnictogen family, has played an important role in the development of phase change and optoelectronic materials [57,58,59]. In particular, chalcogenides based on germanium-antimony-tellurides (GST-PCMs), such as GaSbTe and GaGe_2_Sb_2_Te_5_, display outstanding properties that are a prerequisite for the development of non-volatile memory (NVM) technologies due to their high write and read speeds, reversible phase transition, high degree of scalability, low power consumption, good data retention, and multi-level storage capability [60]. Kao and workers discussed the importance of some Sb-based alloys, viz. Ga_25_Te_8_Sb_67_ and Ga_18_Te_12_Sb_70_, that have a crystallization temperature above 245 °C and activation energy of crystallization greater than 5 eV, and are useful for phase-change memory applications [61]. When combined with halides, they form *inter alia* various single and double perovskites which have been the focus of many studies because of their application in the development of photovoltaics and other areas of optoelectronics. A_3_Sb_2_X_9_ and A_2_AgSbX_6_ are a specific class of such perovskites. A_a_B_b_X_a+3b_ are called rudorfites (A = methylammonium, formamidinium, Cs, Rb, etc.; B = Bi, Sb, etc.; X = I, Br, Cl), and several have been crystallized [59,62,63].

The quantum dots of the all-inorganic Sb-based lead-free double perovskite Cs_2_AgSbX_6_ (X = Cl, Br or I) are examples of an air stable compound that displays strong blue emission with photoluminescence quantum yields of 31.33% [64]. The mixed metal ⟨111⟩-oriented layered perovskite, Cs_4_CuSb_2_Cl_12_, incorporates Cu^2+^ and Sb^3+^ into layers that are three octahedra thick. The compound is photo- and thermal-stable, tolerant of humidity and behaves as a semiconductor with a direct bandgap of 1.0 eV. Its conductivity is an order of magnitude greater than that of the widely studied MAPbI_3_ (MA = methylammonium) perovskite [65].

Apart from the above, many crystal structures have been reported in which antimony plays a prominent role in the development of their structural stability and functionality. It appears that in many cases that covalently bound Sb can act as a pnictogen bond donor to form non-covalent interactions, a driving force for self-assembly. Our search and analysis of the structures deposited in the ICSD and CSD databases suggest that most of the inter- and intra-molecular bonding interactions emerge upon attraction between bound Sb in molecules and halides in the interacting partner entities. The intermolecular bond distances associated with them are often less than, but sometimes marginally greater than, the sum of the van der Waals (vdW) radii of the bound atomic basins. Primary and secondary non-covalent interactions (viz. hydrogen bonding and halogen bonding) are often present, and their interplay with pnictogen bonding drive the packing and hence the overall geometry of the crystal lattice.

## 3. Inter- or Intramolecular Bond Distance and Less Than the Sum of the van der Waals Radii Concept

Our attempts in rationalizing the possible occurrence of a stibium bond hinges on a geometric criterion: whether the inter- or intramolecular distance between covalently/coordinately bound pnictogen atom Pn (viz. Pn = Sb) and a Lewis base(s) D in a crystal lattice, Pn···D (D = an electron density donor, such as O, N, a halogen anion, etc.), is less than (or even slightly greater than) the sum of the van der Waals radii of the bound atomic basins. If the first attribute is met, we recognize the Pn···D link as a likely stibium bond, consequent on the overlapping of atomic domains takes place. In the case of the other attribute, care has to be exercised, since there are numerous occasions in which the inter- or intra-molecular distance, Pn···D, is larger than the sum of the vdW radii of the non-bonded atoms that are in close proximity. In this case, we have taken into consideration crystal systems where the Pn···D distance can exceed the vdW radii sum by several tenths of an ångstrom. This is quite justifiable according to Politzer and coworkers [66], as well as others [67,68], given that the values proposed for the vdW radii of atoms have a typical uncertainty of ±0.2 Å; hence “less than the sum of the vdW radii” concept will necessarily miss a significant number of non-covalent interactions if treated as a strict criterion to identify a non-covalent interaction in molecular entities. The reason is that hard sphere models with spherical symmetry of atoms were considered in the determination of vdW radii of atoms, which differ from one study to the other depending on the approximations invoked in their determination. The underlying reason of course is that the charge density profile of atoms in molecules is anisotropic, and hence the “vdW radius” of an atom in a molecular entity is likely to vary between molecular entities. 

Table 1, for instance, lists the vdW radii of some selected atoms that we invoke throughout this overview, showing that the Bondi’s radius for a given atom type is different from those proposed by others. Since the specific vdW radius of an atom in different molecular entities is unknown, we use the same radius of an atomic domain to examine the “less than sum of the vdW radius concept”, regardless of the lattice systems examined. While “an interatomic distance that is greater than the sum of the respective vdW radii by even several tenths of an ångstrom may still correspond to a non-covalent interaction” [66], caution needs to be exercised to validate the interaction by simultaneously examining attributes like its directional features and the nature of the polarity of the bound atomic basins. Otherwise, such an assignment could be misleading. In our discussion below, we use the vdW radii of atoms proposed recently by Alvarez [68].

## 4. Directionality of Inter-/Intra-Molecular Interactions 

**Scheme 1:**
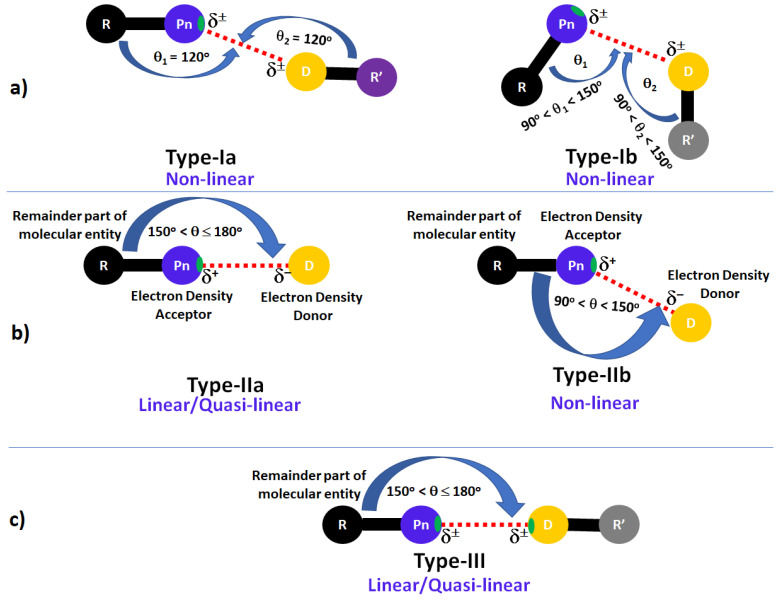
Illustration of (**a**) Type-I, (**b**) Type-II and (**c**) Type-III topologies (geometric) of noncovalent bonding interactions. *θ*’s represents the angle of interaction between the interacting atomic domains, and Pn, D, R and R’ refer to the covalently/coordinately bound pnictogen atom, the interacting atomic domain (generally nucleophilic), and the remaining part of the molecular entities associated with Pn and D atomic basins, respectively. δ± signifies the local polarity (positive or negative), and the small region on atom Pn along R–Pn σ-bond extension colored in green indicates an electron deficient region (a σ-hole).

To maximize the integrity of our identification and subsequent characterization of pnictogen bonding in the examined crystal systems, we simultaneously examined the angle of interaction between the noncovalently bound atomic basins. We did so to see whether the angle *θ* of approach of the electrophile, *θ* = ∠R–Pn···D, associated with the bound Sb atom in a molecular entity forms either a linear, a quasilinear or a bent (non-linear) interaction with the atomic domain in the partner molecular entity. At the same time, we inspected whether the interacting atomic domain D was electrophilic or nucleophilic. As such, Type-I interactions (Scheme 1a), which can be further classified as (left) Type-Ia (*trans*) and (right) Type-Ib (*cis*), appear when the regions of interacting atomic domains in molecular entities both carry either positive or negative local polarity, with *θ*_1_ = ∠R–Pn···D < 120° and *θ*_2_ = ∠R′–D···Pn < 120° for the former and 90° < *θ*_1_ < ∠R–Pn···D < 150° and 90° < *θ*_2_ < ∠R′–D···Pn < 150° for the latter interactions, where R and R′ are the remaining part of the molecular entities associated with Pn and D, respectively. These are non-linear interactions, and hence, should be regarded as non-directional. 

Directional interactions are linear or quasi-linear, and thus, generally follow a Type-IIa topology of bonding. Hence, when the angle of approach *θ* = ∠R–Pn···D = 180°, we call the interaction linear, and when it deviates from linearity such that 150° < *θ* < 180° (Scheme 1b, left), we refer to the interaction as quasi-linear. In either case, the electrostatic surface of covalently/coordinately bound atom Pn conceives an electrophilic region along the extension of the R–Pn covalent or coordinate bond, and D is a Lewis base (such as N in NH_3_, O in OH_2_, and F in HF). However, when 90° < *θ* < 150°, and Pn conceives a positive site, we recognize the interaction it forms as Type-IIb (Scheme 1b, right). Both Type-IIa and Type-IIb interactions are of coulombic origin. 

Type-III interactions (Scheme 1c) originate when the angle of interaction follow a Type-IIa topology of bonding, yet a portion of the interacting atomic domains are either both positive or both negative polarities. This classification scheme has been discussed elsewhere [22,49,50,72].

## 5. The *σ*-Hole and *π*-Hole Concepts, and Their Relationship with Pnictogen Bonds

A *σ*-hole is defined as a region of charge density deficiency, compared to the remainder of the bound atom, on the electrostatic surface of atom A that appears along the outer extension of the R–A covalent bond [73,74]. It can be positive, or negative, or even neutral [22,75]. For instance, a negative *σ*-hole can be observed on the electrostatic surface of atom F along the H–F bond extension of a hydrogen fluoride molecule [76], while a positive *σ*-hole can be seen on the surface of hydrogen halides HX (X = Cl, Br, I) [72,77]. The strength of the *σ*-hole on A is determined by the electronegativity and electron-withdrawing abilities of R, as well as the electronegativity and polarizability of A. Specifically, iodine in HI has a stronger *σ*-hole on its electrostatic surface compared to that of X in HX (X = F, Cl, Br) [72,78]. Similarly, the strength of the positive *σ*-hole on F in CN–F should be weaker than those of CN–X (X = Cl, Br, I), and the strength varies in the order CN–F < CN–Cl < CN–Br < CN–I. This trend is also true for systems with an arene moiety, for example, the strength of the positive *σ*-hole on F in C–F of C_6_F_6_ is weaker than those of C–X (X = Cl, Br, I) in C_6_X_6_, and varies in the order C–F < C–Cl < C–Br < C–I [72]. 

A *σ*-hole centered inter- or intra-molecular interaction involving a positive *σ*-hole on the pnictogen atom along the R–Pn bond extension should be referred to simply as a pnictogen bond [50]. Hypervalent atoms in molecules have more than one σ-hole. For instance, the P in phosphorous trihalides PX_3_ (P = F, Cl, Br, I) possesses three σ-holes, each along the extension of the X–P bond [50]. Similarly, C in CX_4_ (X = F, Cl, Br, I) possesses four σ-holes, each along the extension of each of the four X–C bonds [22,73,76,79]. 

On the other hand, an inter- or intra-molecular interaction may be regarded as a *π*-hole centered pnictogen bond when the pnictogen atom in molecular entities features a *π*-hole on its electrostatic surface and has the ability to engage attractively with a negative site in a neighboring molecule, or a site that has an electron density different to that of the *π*-hole, thus providing stability to the geometry of the resulting structure. A *π*-hole may be defined as an electron deficient region on the surface of a molecular entity; it may appear on the electrostatic surface (centroid region) of a delocalized arene moiety (e.g., C_6_H_6_, C_6_X_6_), on the surface of an atom (e.g., N in NO_3_^−^) or on the central portion of a delocalized bond in a molecular entity (e.g., C≡C and C≡N bonds in HCCH and HCN, respectively). So, analogous with the *σ*-hole, a *π*-hole can be positive or negative. 

A *σ*-hole interaction in a chemical system is generally observed to be directional, whereas a *π*-hole interaction is non-directional.

## 6. Model Systems and Computational Approaches 

As indicated already above, the crystal systems presented in the following sections were retrieved either from the CSD [56] or the ICSD [54,55]. We show, in some cases, that the bound antimony atom in these molecular entities has a positive site, and therefore, is capable of interacting with a negative site of a partner molecular entity. This phenomenon is either wholly or partially responsible for the overall geometric architecture and stability of the resulting crystal lattice. 

The characterization of the positive and negative sites on the surface of molecular entities was made possible by analyzing the electrostatic potentials. We did so by energy-minimizing the geometry of some selected isolated molecular entities in the gas phase. For this, we used the Gaussian 16 program package [80]. The Density Functional Theory at the *ω*B97XD [81] level and Møller–Plesset’s second-order perturbation theory (MP2) [82] were used. Depending on our interest, and for reasons discussed below, basis sets such as Aug-CC-pVTZ or def2-TZVPD were chosen. These were obtained from the basis set exchange library [83,84]. The calculation of the MESP [85] was done using the wavefunctions generated on the fully relaxed geometries of the monomeric entities. For this, AIMAll [86] and Multiwfn [87] codes were used. Geometry analysis, drawing of molecular entities/crystals, and isosurface plots were performed using the Mercury 4.0 [88]/Gaussview 5.0 [89] and VMD [90] suite of programs, respectively.

The isoelectron density envelope on which to calculate the electrostatic potential is quite arbitrary [23,75,77,91,92,93] and can be computed at any contour level, such as 0.001, or 0.0015, or 0.002 a.u. (electrons bohr^−3^) of the total electronic density function *ρ*(*r*). The choice of a 0.001 or 0.002 a.u. envelope was suggested by Bader and co-workers [94,95] and others [96] because it was felt that such an envelope encompasses at least 95% of the electronic charge and should yield physically reasonable molecular “dimensions”. For fluorinated systems, an isodensity envelope greater than 0.001 a.u. is recommended, since this particular envelope often fails to provide insight into the correct nature of surface potential extrema [22,49,50]. 

The promolecular charge density based isosurfaces were calculated within the promolecular framework of Independent Gradient Model (IGM) [97,98]. We did so because we were interested in the identification and subsequent characterization of Sb-centered pnictogen bonding within or between interacting monomer entities responsible for the crystals investigated, and to cross-check the reliability of these interactions emanated using intermolecular bond distances, directionality, and MESP model descriptions. Within the framework of IGM, promolecular atomic electron densities are summed, and the associated atomic gradients do not interfere. This can be achieved by using absolute values to sum the atomic gradients and by rejecting any electron gradient contragradience feature. The resulting total gradient |∇*ρ*^IGM^| is an upper limit of the true gradient, and the difference between them, *δg*, quantifies the net electron density gradient collapse due to interactions. Ultimately, this means that the *δg* descriptor identifies the presence of opposite signs in the components of the total electron density gradient |∇*ρ*(r)| due to interactions. IGM thus has the capacity to automatically separate intra- and inter-fragment interactions in a molecular entity, and that this can be plotted in 2D or in 3D (isosurfaces) to reveal the presence of inter- or intramolecular interactions between interacting atomic basins. The 3D shape of the isosurface volumes can be utilized to infer localized or delocalized interactions between interacting domains. The colors of these isosurface volumes, in blue and green, represent strong and weak attractions, respectively, while red represents a repulsive interaction. Hereafter, we refer to this approach as IGM-δ*g*.

## 7. The Molecular Electrostatic Potential and Characterization of σ- and π-Holes in Molecular Entities 

The extrema of potential on the surfaces of molecular entities appear in two different flavors [27,28,29,30,99,100], i.e., the local most minimum of potential, *V_S,min_*, and the local most maximum of potential, *V_S,max_*. They may be positive, or negative, or neutral in specific regions on the surface of a molecular entity depending on the extent of electron density depletion or electron density accumulation. Lone-pair regions on the surface of a covalently bound atom, or on the surface of an anion, should feature a *V_S,min_*, since such regions have high electron densities.

The positive and negative signs of *V_S,min_* or *V_S,max_* generally refer to regions that are electrophilic and nucleophilic, respectively. So an electrophilic region can be recognized when *V_S,min_* > 0 or *V_S,max_* > 0. Similarly, a nucleophilic region can be identified when *V_S,min_* < 0 or *V_S,max_* < 0. The magnitude of *V_S,min_*, or *V_S,max_* determines the strength of the potential; the larger the magnitude of *V_S,min_*, or *V_S,max_*, the stronger the nucleophilicity, or electrophilicity, of the region concerned. For instance, *V_S,max_* was observed to be positive and larger on the surface of the I than on X (X = F, Cl, Br) along the extension of the H–X and C–X covalent bonds in HX and CX_4_ molecules; hence the electrophilicity of covalently bound I along the H–I and C–I bond extension is the stronger when compared to that of Br, Cl, F in each of the two families of molecular entities. Since *V_S,max_* on X in these molecules appears along the extension of the H–X/C–X σ-bonds, it describes the sign and magnitude of the *σ*-hole on X. The concept is transferable to other covalently bound atoms; thus a positive or negative σ-hole can be found on the surfaces of a covalently bound triel, tetrel, chalcogen, pnictogen and halogen atoms, among others, depending on whether *V_S,max_* is positive or negative along the outer extension of the covalent bond. 

An attractive coulombic interaction between two atomic basins (intermolecular, or intramolecular) may be recognized when a region of an atom or fragment in a molecular entity with a positive *V_S,min_* (or *V_S,max_*) is in close proximity to that with a negative *V_S,min_* (or *V_S,max_*) on the same, or a neighboring, molecular entity. 

A *σ*-hole interaction between two atomic basins (intermolecular, or intramolecular) may be recognized when a positive *V_S,max_* on atom A along the R–A bond extension is in close proximity to a negative site described by a negative *V_S,min_* or *V_S,max_*. When atom A = Pn conceives a positive *V_S,max_*, the attractive engagement is a *σ*-hole centered pnictogen bound interaction, or simply a pnictogen bond.

Similarly, a *π*-hole centered intermolecular or intramolecular interaction between two atomic basins may be recognized when a positive *V_S,min_* or *V_S,max_* centered on the p-orbital of atom, a bond, a delocalized ring, or on a fragment, in a molecular entity is able to attract a negative site described by a negative *V_S,min_* or *V_S,max_* on another similar, or different, molecular entity. When a positive *π*-hole is found on a pnictogen atom in a molecular entity, and if it is a capable for attracting a negative site on the same, or a different molecule, the resulting interaction between them is a consequence of *π*-centered pnictogen bonding. 

There are many reports of *σ*-hole and *π*-hole interactions forming the basis of the stability of numerous chemical systems. Some of them, for instance, include discussions about their similarities and differences, as well as the controversies and misconceptions surrounding this concept [51,73,101,102,103,104,105,106].

## 8. Illustrative Crystal Systems

As we show below, we also observed that Sb in molecular entities can cause the formation of pseudo-covalent bonds when it finds itself in close proximity to a negative site in another molecular species. It has the capability to accept electron density from lone-pairs centered on O, N, F, Cl, Br, and I in other molecules. Antimony trihalides SbX_3_ (X = F, Cl, Br, I) are probably the most simplest, best examples that feature the ability of Sb to form non-covalent interactions with negative sites on a neighboring molecule (*vide infra*). The ability of covalently bound Sb to form very strong pnictogen bonds upon its attractive engagement with halide anions has been recognized, with the energy of these interactions reaching the lower bound of covalent bonding energies [107]. It was argued that such interactions comprise significant covalent character, even though these were sometimes thought to be essentially electrostatic interactions. 

### 8.1. Antimony Trihalides

**Figure 1:**
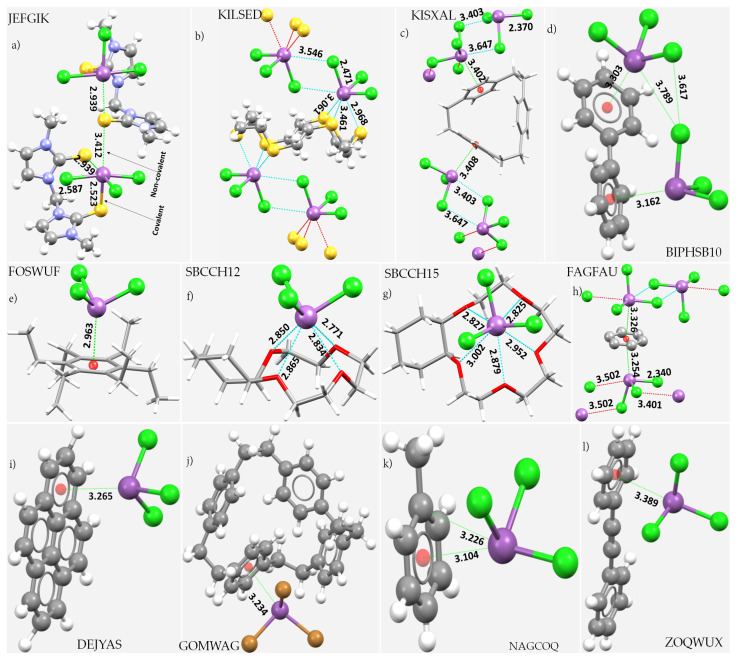
Some selected examples (with CSD codes in capital letters) illustrating the possibility of Sb···X pnictogen bonding interactions formed by covalently bound Sb in SbCl_3_ and SbBr_3_ upon attractive engagement with a variety of interacting molecules containing pnictogen bond acceptors X, where X refers the halogen derivatives, O, S, and π-density. Selected Sb···X intermolecular distances and the Sb–X covalent/coordinate bond distances (in Å) are shown for most cases, indicating clear evidence of the difference in distances between Sb···X and Sb–X. The centroid of the arene moiety (tiny sphere in red) to Sb distances in several cases are also shown in Å. Atom type is shown where appropriate. The compounds are (**a**) SbCl_3_mbit (mbit = bidentate 1,1’-methylene-bis(3-methyl-2H-imidazole-2-thione) [108]; (**b**) SbCl_3_.18S_6_ (18S_6_ = the crown ether 1,4,7,10,13,16-hexathiacyclooctadecane) [109]; (**c**) (C_24_H_24_)(SbCl_3_)_2_ (C_24_H_24_ = [2.2.2]paracyclophane) [110]; (**d**) biphenyl bis(antimony trichloride) [111]; (**e**) (C_18_H_30_)SbCl_3_ (C_18_H_30_ = hexaethylbenzene) [112]; (**f**) (cyclohexo-12-crown-4)SbCl_3_ [113]; (**g**) (cyclohexo-15-crown-5)SbCl_3_ [114]; (**h**) (benzene)(SbCl_3_)_2_ [115]; (**i**) pyrene bis(trichloro-antimony) [116]; (**j**) [2.2.2.2]-Paracyclophane tribromo-antimony [117]; (**k**) trichloro-antimony toluene [118]; (**l**) tetrakis(trichloro-antimony) 1,2-diphenylethyne [119].

**Figure 2:**
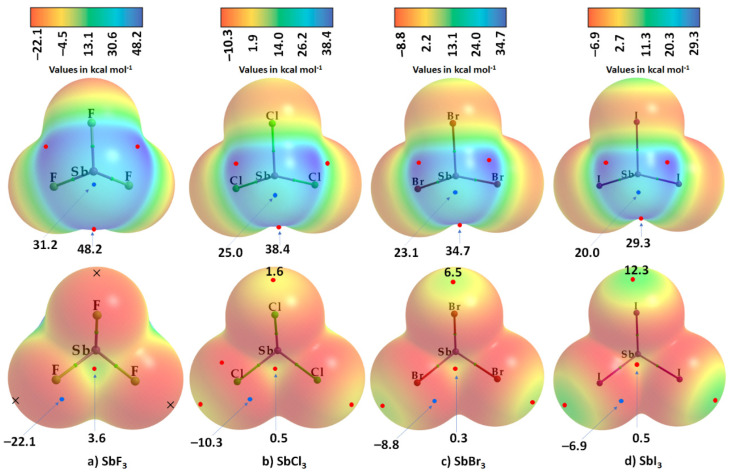
MP2(full)/def2-TZVPPD calculated 0.001 a.u. isoelectron density mapped potential on the electrostatic surfaces of SbX_3_ (X = F, Cl, Br, I) molecules: (**a**) SbF_3_; (**b**) SbCl_3_; (**c**) SbBr_3_; (**d**) SbI_3_. Selected *V_S,max_* and *V_S,min_* values in kcal mol^−1^ are shown, which are the local most minimum and maximum of potential (tiny circles in blue and red), respectively. Two views of the MESP graph for each molecule are displayed. (**Top**): Covalently bound Sb faces the reader. (**Bottom**): The triangularly arrayed X atoms face the reader. The crosses (×) shown along the extensions of the Sb–F bonds highlight the absence of a σ-hole on F, indicating that F is entirely negative.

In the various examples provided in Figure 1, we mainly focus on highlighting the intermolecular bonding modes of SbX_3_ and a few other compounds containing Sb. As can be seen, the SbX_3_ molecules have a trigonal pyramidal molecular geometry. In order to understand the chemical reactivity of the various constituents of SbX_3_ that are responsible for the intermolecular interactions in the crystals, we focused on examining the nature of the electrophilic and nucleophilic regions on the electrostatic surfaces of isolated SbX_3_ molecules. We did so by exploring the MESP at the MP2(full)/def2-TZVPPD and ωB97XD/def2-TZVPPD levels of theory. Our results for the local maxima and minima of potential on the electrostatic surface of the SbX_3_ molecules are shown in Table 2. Figure 2a–d shows the MESP graphs for the isolated SbX_3_ molecules.

Based on a simple Lewis model, SbX_3_ should be a *C*_3*v*_ molecule with a lone pair at the apex of the pyramid. The ground state electronic configuration of Sb^3+^ is [Kr] 4d^10^ 5s^2^. Despite the (formal) localization of the lone pair in an *s* orbital, the lone pair in many Sb^3+^ compounds is stereochemically active. For example, the gas phase structure of SbCl_3_ is trigonal pyramidal (approximately *C_3v_*) as determined by gas-electron diffraction with a Cl–Sb–Cl angle of 97.2(9)° [120], in agreement with the rotational constants obtained from microwave spectroscopy [121]. Salts of SbF_4_^−^, SbCl_4_^−^ and Sb_2_F_9_^3^^−^ have large quadrupole splitting in their ^121^Sb Mössbauer spectra [122]. This, and the ^121^Sb Mössbauer data of other Sb^3+^ compounds (such as Sb_3_F_10_^−^, Sb_4_F_13_^−^ and Sb_5_F_19_^4^^−^), are consistent with a stereochemically active 5s electron pair in (using the nomenclature of VSEPR) an AX_6_E environment, featuring a mono-capped octahedron with the lone pair in an apical position [123]. Quoting others, it has been pointed out [124] that in the solid state Sb^3+^ usually has *n* ligands at relatively short distances on one side of the ion (hemisphere I) and *m* ligands on the other side (hemisphere II) at significantly longer distance, often of the same order as the sum of the vdW radii of antimony and the atom of the hemisphere II ligand with which it interacts. This is attributed to the stereochemical effect of the lone pair on Sb^3+^ directed towards hemisphere II. Moreover, an analysis of the Voronoi-Dirichlet (VD) polyhedra of a large number of Sb^3+^ compounds showed that Sb^3+^ (unlike compounds of Sb^5+^) is very often displaced from the center of gravity of its VD polyhedron, indicative of the presence of a stereochemically active lone pair [124].

There are cases in which the 5s electron pair appears to have no stereochemical influence. Thus, in (NH_4_)_2_Sb_2_Br_12_, Sb^3+^ is *O_h_* and the Br–Sb–Br angles are insignificantly different from 90°, while the Sb–Br bonds are significantly lengthened [125]. This may be analogus with phosphorus(III) compounds of the type Ar_2_PX (Ar = 2,5-bis(trifluoromethyl)phenyl, X = Br, Cl) that feature a stereochemically inactive lone pair, with P^3+^ displaying an approximately octahedral geometry [126].

The electron density associated with the stereochemically active lone pair in NX_3_ and PX_3_ were previously reported from evidence of their corresponding MESP plots [49,50], but this is not apparent in the MESP of SbX_3_ (Figure 2). This does not mean that the lone pair is not stereochemically active; it is there, but what is evident is the gradual polarization of charge density from Sb to X as the softness of Sb compared to N [49] and P [50] increases (and its electronegativity decreases) down the group.

Nevertheless, from Table 2, it can be seen that F in SbF_3_ is entirely negative and the *σ*-hole on it is completely neutralized. The trend in the stability of the *σ*-holes along the Sb–X bond extensions in SbX_3_ increases with an increase in the polarizability of X: Sb–Cl (1.6 kcal mol^−1^) < Sb–Br (6.5 kcal mol^−1^) < Sb–I (12.3 kcal mol^−1^) (Figure 2a–d). The lone pair regions on X in the same molecules, described by *V_S,min_*, become less negative as one passes from the lightest to the heaviest member of the halogen family. The most positive regions on the surfaces of the SbX_3_ molecules are identified on Sb along the three X–Sb bond extensions and that the stability of Sb’s *σ*-hole follows the order SbF_3_ > SbCl_3_ > SbBr_3_ > SbI_3_. The central region of the triangular face formed by the three halogens is described by a weakly positive *V_S,max_*, and is surrounded by negative potentials. These features of the electrostatic potential, elucidated by ab initio MP2(full) calculations, are very similar to those that arise from DFT-based ωB97XD calculations (cf. Table 2). 

The existence of the electrophilic sites on Sb and the nucleophilic sites on X in SbX_3_ (X = Cl, Br) explains that the attractive interactions that led to the development of the Sb···X intermolecular bonding interactions that were observed in the crystal geometries shown in Figure 1 were coulombic in nature.

### 8.2. Tetramethyl-Antimony Iodide

**Figure 3:**
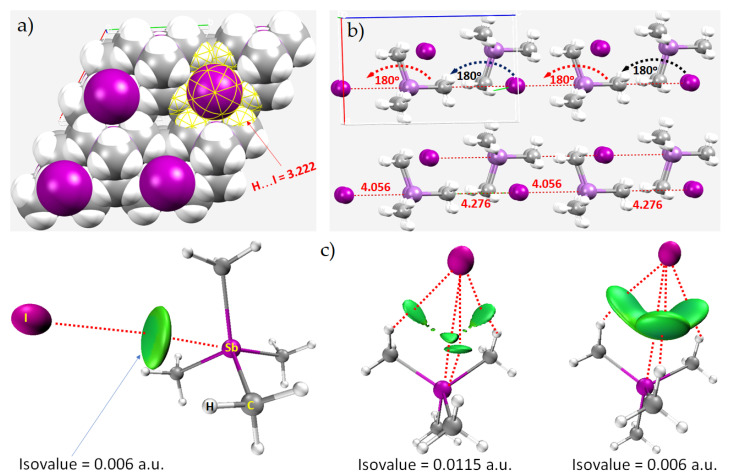
(**a**): The space-filling model of the unit-cell of [(CH_3_)_4_Sb]I (CSD ref. code: SUCZEV). (**b**) The 2 × 2 × 2 supercell structure of the same crystal, with selected intermolecular distances and angles shown. (**c**) The IGM-δ*g* based isosurface plots for the ion-pair [Sb(CH_3_)_4_]^+^[I]^−^, obtained on the crystal geometry. Selected bond lengths and bond angles are in Å and degree, respectively. Dotted lines represent intermolecular interactions. Atom type is marked in (**c**).

Tetramethyl-antimony iodide salt [(CH_3_)_4_Sb]^+^[I]^−^ is a good example of a solid state structure featuring charge-assisted pnictogen bonding in a sterically crowded environment; see Figure 3a [127]. We did not perform a MESP calculation for the system, since the polarity of the molecular domains responsible for the crystal lattice is quite apparent. The methyl hydrogen atoms of the three nearest (CH_3_)_4_Sb^+^ moieties of the crystal are non-covalently bound with iodide anions through charge-assisted C–H···I hydrogen bonds. Six such prominent interactions are formed with I^−^. They are all equivalent and the angle of attack for the formation for each of these and the corresponding intermolecular distances are ∠C–H···I = 147.4° and 3.222 Å, respectively. Apart from this, [(CH_3_)_4_Sb]I features charge-assisted Type-IIa C–Sb···I pnictogen bonds (*r*(Sb···I) = 4.056 and 4.352 Å) and Sb–C···I tetrel bonds (*r*(C···I) = 4.276 Å) that are significantly longer than the hydrogen bonds, and are directional interactions (∠C–Sb···I = 180° and ∠Sb–C···I = 180°). The long intermolecular distances are not unexpected, since the sum of the vdW radii for C and Sb (*r*(C)_vdW_ = 1.77 Å and *r*(Sb)_vdW_ = 2.47 Å) is larger than that of I and H (*r*(I)_vdW_ = 2.04 Å and *r*(H)_vdW_ = 1.20 Å). 

Our observation is in line with that of the authors of [127], in that the coordination around antimony is distorted tetragonal pyramidal, and the tetrahedral coordination of the ions in the structure results in the formation of a wurtzite-type structure with antimony-iodine distances of 4.06 and 4.35 Å. Although the non-covalent bonding features that occur in the crystal constitute *σ*-hole interactions, the involvement of the *σ*-holes on H and Sb in the long-range interactions cause the formation of a layer-type molecular framework in the crystal (Figure 3b).

Our contention that C–H···I hydrogen bonds and C–Sb···I pnictogen bonds exist in the extended crystal is given credence by the IGM-δ*g* based isosurface plots shown in Figure 3c, obtained at three different isovalues. The circular volume (green) in the graph on the left occurs between Sb and I atomic basins, whereas the isosurfaces that occur between the two molecular entities are illustrated in the central and right side of the graph. These features suggest possible attractive interactions between bound atomic basins.

### 8.3. The Co-Crystal of Antimony Trihalide and Molecular Sulfur

**Figure 4:**
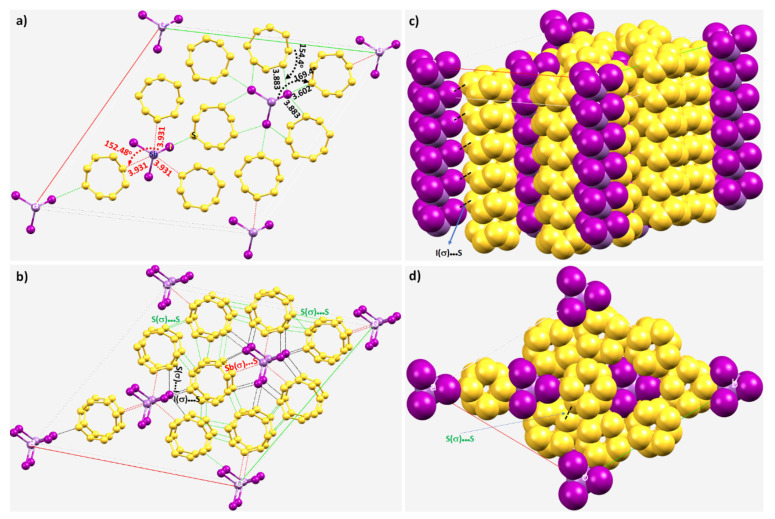
(**a**) The unit cell of the adduct between antimony triiodide and molecular sulfur, SbI_3_:3S_8_ (ICSD ref. code. 14200). (**b**) Illustration of the 1 × 1 × 2 supercell structure of the adduct, showing the network of various intermolecular interactions involved in the formation of the crystalline material. (**c**,**d**) The side and top views of the nature of the packing in the SbI_3_:3S_8_ crystal. Selected bond lengths and bond angles are in Å and degree, respectively. Dotted lines represent intermolecular interactions. Atom type is marked in (**a**).

The addition compound SbI_3_:3S_8_ crystallizes in the *R*3*m* space group [128]. The structure is stabilized by many non-covalent interactions. As noted in the study, each iodine atom of the SbI_3_ unit is attached to a S atom of an S_8_ moiety with a Sb–I···S distance of 3.60 Å and an ∠Sb–I···S angle of 169.4°. Each I-bound Sb also links with three nearest neighbor S sites of S_8_ crown-shaped molecules, forming Sb–I···S intermolecular contacts, with I–Sb···S = 3.391 Å and an ∠I–Sb···S angle of 152.5°. These bonding features can be inferred from Figure 4. 

Our calculation of the MESP of SbI_3_ and S_8_ at the ωB97XD/Aug-CC-pVTZ level of theory provides a more in-depth understanding of the bonding modes in this compound than that of the original study. 

**Figure 5:**
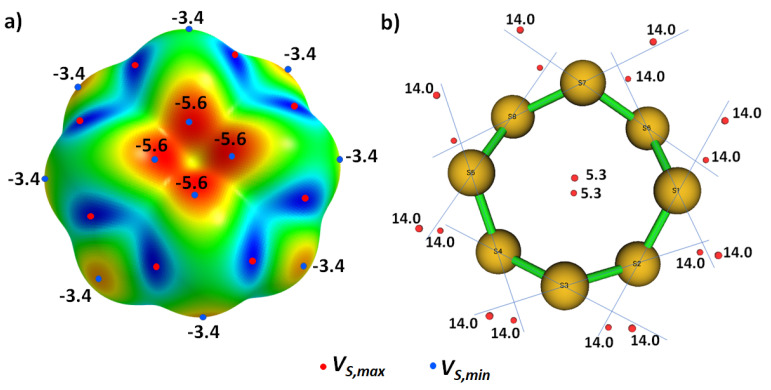
(**a**) ωB97XD/aug-cc-pVTZ computed 0.0015 a.u. isoelectron density mapped potential on the electrostatic surface of the S_8_ molecule. (**b**) Illustration of the molecular framework, with only the local most maximum of potential (*V_S,max_*) along the outer extensions of the S–S covalent bonds. Values of the local most minima of potential (*V_S,min_*) on the electrostatic surface of the S_8_ molecule is shown in (**a**). Values of *V_S,max_* and *V_S,min_* are in kcal mol^−1^.

The MESP graph of S_8_, together with all selected local most maxima and minima of potential on the electrostatic surface of the molecule, are shown in Figure 5. The molecule has eight pairs of equivalent maxima of potential (*V_S,max_* = 14.0 kcal mol^−1^ each) that arise along each S–S bond extension, slightly off the *σ*-axis; each maximum is associated with a *σ*-hole on S (cf. Figure 5a,b). The centroid region of the molecule features two maxima of potential, one on the top and one at the bottom surface; they are equivalent (*V_S,max_* = 5.3 kcal mol^−1^ each) and weaker than the *σ*-hole on S (Figure 5b). Each of these electrophilic regions on covalently bound S has the potential for chalcogen bonding interactions when in close proximity of a negative site. By contrast, the lateral portion of each covalently bound S atom in S_8_ has a negative potential (Figure 5a). These loci of negative potential toward the interior of the S_8_ ring are more negative (*V_S,min_* = −5.6 kcal mol^−1^) than those on the exterior surface of the ring (*V_S,min_* = −3.4 kcal mol^−1^). 

The authors of the original study [128] suggested the presence of intermolecular bonding between I and S sites, basing this conclusion on the intermolecular distance between I and S atoms, and within the conceptual framework of Lewis structures of chemical bonding. In particular, they observed that each iodine atom has two pairs of sulfur neighbors with I···S separation distances lying between 3.78 and 3.88 Å. The stability of the structure was attributed not only to these I···S charge transfer interactions, but also to some extent to Sb···I interactions. However, it was not clear whether these interactions are halogen bonds or chalcogen bonds in the first case, or halogen bonds or pnictogen bonds in the second case, since these bonding terminologies were not coined in 1963. 

With the computational tools now available, we are able to more precisely elucidate the nature of these interactions. Our analysis indicates that each I site in SbI_3_ is involved in three S···I interactions. Two of them are equivalent chalcogen bonds, with *r*(S···I(Sb)) = 3.883 Å. They are a result of attractive coulombic interaction between the lateral negative portions of bound I atoms in SbI_3_ and the axial positive portions of the S atom along the S–S bond extensions in S_8_. They are relatively far from being linear, Type-IIa (∠S–S···I(Sb) = 154.4°). The third S···I interaction is a quasi-linear Type-IIa (Sb)I···S halogen bond, since the σ-hole on I along the Sb–I bond extension is in an attractive engagement with the negative site on S in S_8_ (Figure 5a). 

Similarly, each covalently bound Sb site along the I–Sb bond extension in SbI_3_ is bound to the nearest negative sites localized on three S atoms of the three nearest S_8_ molecules, forming three equivalent Type-IIa I–Sb···I bonding interactions (*r*(Sb···S) = 3.391 Å and ∠I–Sb···S = 152.5° for each). These interactions have the characteristics of quasi-linear pnictogen (stibium) bonds. 

**Figure 6:**
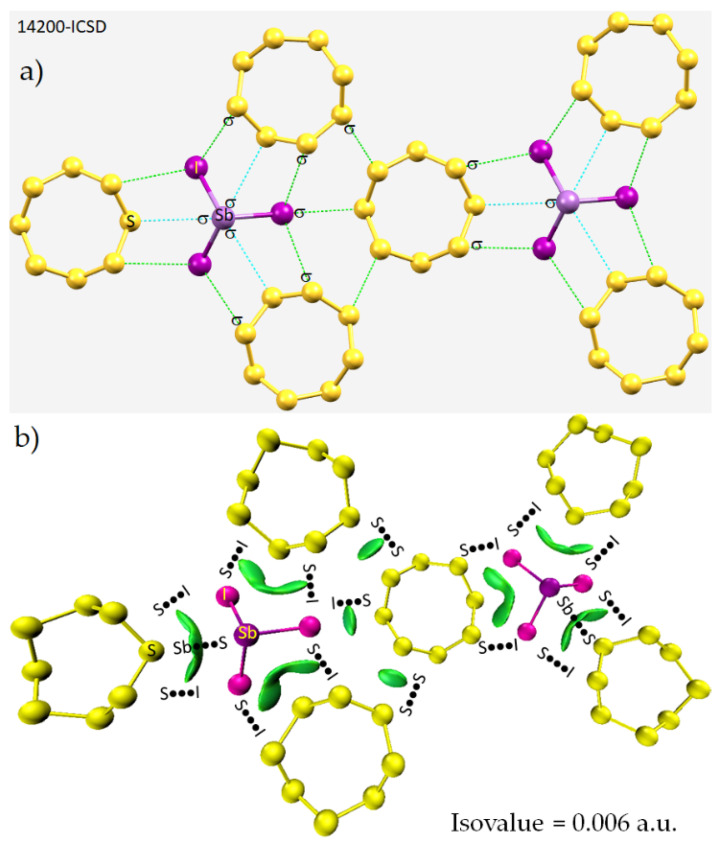
Illustration of various *σ*-hole centered intermolecular interactions in the crystal structure the adduct SbI_3_:3S_8_. Some molecules in the cell have been deleted for clarity. Shown in (**b**) are the IGM-δ*g* based isosurface topologies (green) explaining the intermolecular bonding interactions between various interacting atomic domains. The symbol “*σ*” on some atoms in (**a**) signifies the presence of a *σ*-hole, and the intermolecular interactions are represented by dotted lines in cyan or green. Atom type is shown in each case.

Other than the three predominant interactions noted above, the crystal features numerous S–S(*σ*)···S type interactions that are quasi-linear. The deviation from linearity is probably because the maximum of potential on the electrostatic surface of S (*σ*-hole) is off the S–S bond axis (Figure 5b). Nevertheless, we observed Type-IIa S–S(*σ*)···S interactions between the nearest S_8_ rings, featuring interaction distances of 3.469, 3.484, and 3.755 Å, corresponding to ∠S–S(σ)···S of 152.5, 161.9, and 169.5°, respectively. Clearly, the extensive network of Type-II noncovalent interactions, together with numerous Type-I S···S interactions between the interacting units (not shown), determine the shape of the crystal, and can be rationalized from Figure 6a,b. Our characterization of intermolecular bonding interactions in SbI_3_:3S_8_ are supported by the IGM-δ*g* based isosurface topologies shown in Figure 6b, in which, there are clear isosurface volumes between bound atomic basins that are bonded with each other. 

**Figure 7:**
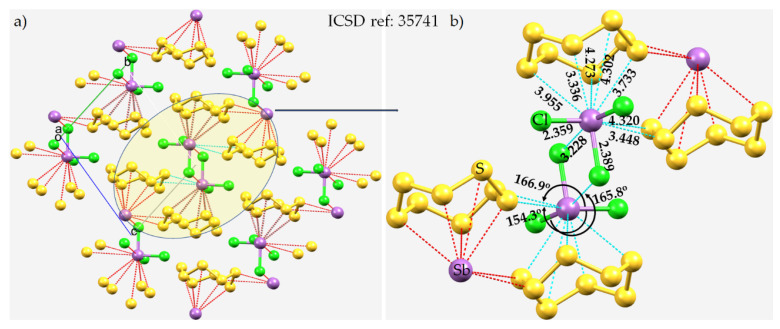
(**a**) The 2 × 2 extended crystal structure of the adduct SbCl_3_.S_8_. Hanging contacts are shown by dotted lines in red. The portion of the crystal in (**a**) encircled by an ellipse is shown in (**b**). Bond lengths and bond angles are in Å and degrees, respectively. Not all the Cl···S halogen bound and the S···Cl chalcogen bound contacts are shown. Atom type is marked in (**b**). The long arrow in black refers to the cluster that is encircled in (**a**).

Another adduct between an antimony halide and molecular sulfur is SbCl_3_.S_8_; see Figure 7. It crystallizes in the triclinic P-1 space group [129]. The unit-cell contains two SbCl_3_ and two S_8_ molecules, and the crystal is zero-dimensional [130]. Sb in each SbCl_3_ molecule links with several S sites of the crown-shaped S_8_ ring, as well as with the negative sites on Cl in close proximity (see dotted lines in Figure 7a). Compared to the formal Sb–Cl bond distances (*r*(Sb–Cl) = 2.60–2.34 Å; see Figure 7b) between Sb and Cl which are responsible for the trigonal pyramidal geometry of SbCl_3_, the Sb···S and Sb···Cl intermolecular interactions are much longer (*r*(Sb···S) in the range 3.448–4.320 Å; *r*(Sb···Cl) = 3.228 Å; Figure 7b). Several of them are non-linear and those appearing along the three Cl–Sb extensions are quasi-linear. All these interactions are Sb-centered pnictogen bonds. Additionally, there are Cl···S halogen bonds, and S···Cl and S···S chalcogen bonds in the crystal (not shown). The formation of these intermolecular interactions is in agreement with the electrostatic surface features of the interacting molecules revealed by the MESP model discussed above (see Figure 2b and Figure 4 for SbCl_3_ and S_8_, respectively); together they are responsible for the stability of the SbCl_3_.S_8_ system in the crystalline phase. Theoretically, the SbCl_3_.S_8_ crystal system is thermally stable with a bandgap of 2.88 eV [130], and is thus a semiconducting material.

### 8.4. Antimony Trihalide Crystals

**Figure 8:**
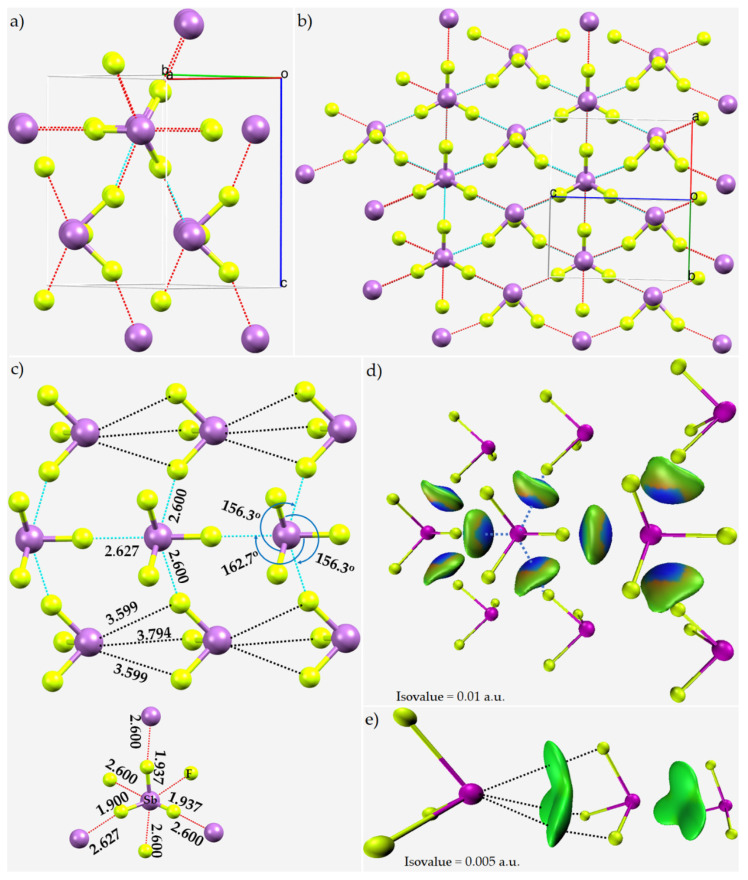
(**a**,**b**) The unit-cell and 2 × 2 extended crystal structures of SbF_3_ (ICSD ref. 16142), respectively; the latter shows a pseudo 3D framework of the system. (**c**) Illustration of the local topology bonding between the SbF_3_ units in the crystal. (**d**,**e**) Representation of the IGM-δ*g* based isosurface topologies between the SbF_3_ units in the crystal. Selected bond lengths and bond angles are in Å and degrees, respectively. Blue and green isosurfaces in (**d**,**e**) represent strong and weak interactions between F and Sb in the crystal, and were calculated at different isovalues. Intermolecular interactions between molecular entities are represented by dotted lines.

To validate the results of the MESP model summarized in Table 2, we examined the structure of SbF_3_, reported in 1970 [131]. The unit cell and 2 × 2 extended crystal structure of SbF_3_ are shown in Figure 8a,b. The dotted lines between the SbF_3_ units represent intermolecular interactions. Each Sb has three close fluorine neighbors with mean Sb–F distances 1.92 Å and each SbF_3_ unit is linked with three fluorine bridges (*r*(Sb···F) = 2.61 Å) to form a pseudo three-dimensional network, with a much-distorted octahedral co-ordination around the antimony atom.

Our MESP calculations, discussed in Section 8.1. Antimony Trihalides above, indicate that the surface of Sb is strongly positive along the F–Sb bond extensions, while F is entirely negative. This suggests that there are three *σ*-holes along the extensions of the three F–Sb bonds. The nature of the electrostatic potential surfaces of Sb and F explains why is there a coulombic attraction between Sb in one SbF_3_ monomeric unit and F in a neighboring unit in the crystal, and why there are three Sb···F intermolecular interactions along the three F–Sb bond extensions in a pair of SbF_3_. They are also directional, with a pair of Sb···F interactions displaying an ∠F–Sb···F = 156.3° and the remaining other has ∠F–Sb···F of 162.7°. These interactions are Sb-centered *σ*-hole interactions and are relatively weaker than the formal Sb–F covalent bonds (bond lengths 2.61 Å versus 1.92 Å; cf. Figure 8c, bottom). However, they are quite strong pnictogen bonds, as attested to by the IGM-δ*g* based isosurface colored bluish-green in Figure 8d.

Other than these interactions, we found a pattern of a parallel arrangement between the SbF_3_ units along the crystallographic *b*-axis, in which the axes of neighboring SbF_3_ units are aligned (Figure 8c, top). In this arrangement, Sb in an SbF_3_ unit faces the center of the triangular face formed by three F atoms in another a neighboring SbF_3_ unit (see the first and third columns of the cluster shown in Figure 8b). These are relatively weak and non-linear Type-Ib pnictogen bound interactions, with *r*(Sb···F) between 3.60 and 3.80 Å and ∠F–Sb···F in the range 131.8–136.7°. They are also observable in the IGM-δ*g* based isosurface plot shown in Figure 8d,e.

The extensive network of pnictogen bonding not only drives the formation of the pseudo 3D framework of the crystalline form of SbF_3_, but also the development of second-order nonlinear optical (NLO) properties [132,133]. In fact, the SbF_3_ system exhibits a powder second harmonic generation (SHG) which is about 5.8 times that of potassium dihydrogen phosphate (KDP), and is an NLO material in the infrared region. This allows excellent transparency in the range of 0.29–12 μm with high thermal stability. The band gap of the material calculated from the UV–vis–near-infrared spectrum was about 4.3 eV [132]. 

**Figure 9:**
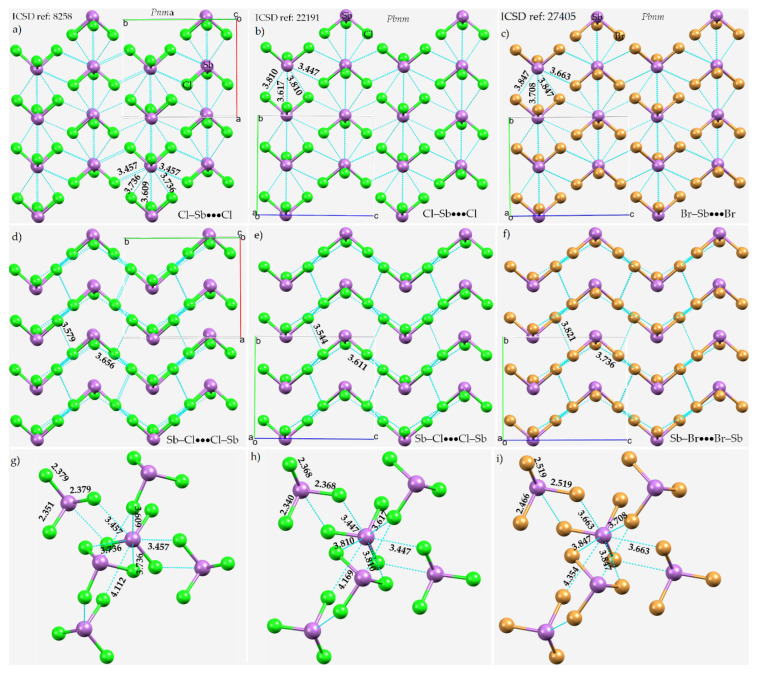
(**a**,**b**) The 2 × 2 extended structures of the SbCl_3_ crystal in the space groups *Pnma* and *Pbnm*, respectively; (**c**) The 2 × 2 extended structure of SbBr_3_ (space group *Pbnm*). In all three cases, Cl–Sb···X (X = Cl, Br) intermolecular pnictogen bonding interactions are illustrated as dotted lines in cyan. (**d**–**f**) Representation of the structures of the same crystal systems, illustrating the presence of X···X (X = Cl, Br) intermolecular contacts. (**g**–**i**) Illustration of the local pnictogen bonding environment around the Sb^3+^ ion in SbX_3_. Selected bond distances are in Å and degrees, respectively.

The 2 × 2 extended cementite crystal structures of SbX_3_ (X = Cl, Br) are shown in Figure 9a–c. The unit-cell for each contains four molecular formula units SbX_3_ crystallized in an orthorhombic space group–as SbI_3_, *vide supra*–SbCl_3_ in *Pnma* [134] and *Pbnm* [135]; SbBr_3_ in *Pbnm* [136]. There are two equivalent Br sites in the structures, forming a shorter and two longer, equivalent, Sb–X bond distances. The structures are zero-dimensional and Sb^3+^ is bound in a distorted T-shaped geometry to three halogen atoms [137,138]. Kang et al. [133] have demonstrated that SbX_3_ (X = Cl, Br) are good mid-IR NLO crystals materials. The DFT+U calculated bandgaps were calculated to be 3.751 and 3.119 eV for SbCl_3_ [138] and SbBr_3_ [137], respectively, suggesting that they are wide band gap semiconductors. 

Our geometric analysis of the structure shown in Figure 9b indicates that Sb in SbCl_3_ is linked to nine Cl neighbors in five surrounding SbCl_3_ molecules. Three of them are formal Sb–Cl bonds expected of the SbCl_3_ molecule. The remaining six are intermolecular Sb···Cl contacts, with bond distances between 3.447 and 4.169 Å. Each of them is less than the sum of the vdW radii of Cl and Sb, 4.29 Å (*r_vdW_*(Sb) = 2.47 and *r_vdW_*(Cl) = 1.82 Å), and are all formed between the positive surface on Sb along the Cl–Sb bond extensions in one molecule of SbCl_3_ and the negative lateral portions of the Cl atom around the Sb–Cl bonds a neighboring molecule(s). Of the six, the three Sb···Cl contacts developed along the three Cl–Sb bond extensions are directional; a pair of bonds with ∠Cl–Sb···Cl = 165.4° are associated with the bond distances of 3.447 Å, and the third one, that has the longest bond distance of 4.169 Å, is associated with an ∠Cl–Sb···Cl of 154.4°. The remaining three Sb···Cl interacts are non-linear (∠Cl–Sb···Cl = 137.9°, 126.2°, and 132.7°). A very similar topology of pnictogen bonding interactions is revealed for the same system crystallized in the space group *Pnma* [134], although the Sb···Cl bond lengths and ∠Cl–Sb···Cl bond angles differ slightly (Figure 9a).

Because SbBr_3_ crystallizes in the same space group, i.e., *Pbnm* [136], the local bonding environment around the Sb ion in SbBr_3_ is very similar to that in SbCl_3_. For instance, we also observed a total of nine Br neighbors around the Sb ion in SbBr_3_; six of them are Sb···Br intermolecular interactions (Figure 9i). Of these six, the three appearing along the Br–Sb bonds are quasi-linear Type IIa interactions and the remaining three, emanating off the Br–Sb *σ*-bonds, are non-linear. In particular, a pair have ∠Br–Sb···Br = 165.3° and a bond distance of 3.663 Å. The third has the longest bond distance of 4.354 Å with an ∠Br–Sb···Br = 155.8°. The remaining three non-linear interactions are associated with ∠Br–Sb···Br of 125.4, 137.1 and 133.4°. We attribute all the quasi- and non-linear Sb···Cl and Sb···Br interactions in the respective crystals of SbCl_3_ and SbBr_3_ to intermolecular pnictogen bonds that follow either a Type-II or a Type-I bonding topology. 

There are extensive Type-I and Type-II Sb–X···X and Sb–X···X–Sb (X = Cl, Br) halogen bound contacts in each of the three crystals discussed above. They are shown in Figure 9d, Figure 9e and Figure 9f for SbCl_3_ (*Pnma*), SbCl_3_ (*Pbnm*) and SbBr_3_ (*Pbnm*), respectively. The Type-I Cl···Cl contacts in SbCl_3_ that occur between the zig-zag sheets are shorter than those within the sheet itself, and hence responsible for the sheet’s development (viz. 3.579 Å versus 3.630 Å in Figure 9d; viz. 3.544 Å versus 3.626 Å in Figure 9e). The opposite trend is seen in SbBr_3_. The analogous Type-I Br···Br contacts are 3.821 Å and 3.758 Å (Figure 9f). The Type-II Sb–X···X contacts in all three crystals are within the zigzag chains; the distances are 3.656, 3.611 and 3.736 Å in SbCl_3_ (*Pnma*), SbCl_3_ (*Pbnm*) and SbBr_3_ (*Pbnm*), respectively. The local pattern of pnictogen and halogen bonding interactions are shown in Figure 9g–i. Clearly, the combination of a 3D network of Type-I and Type-II halogen and pnictogen bonding interactions do not leave the SbCl_3_ molecules in the crystal as zero-dimensional. 

**Figure 10:**
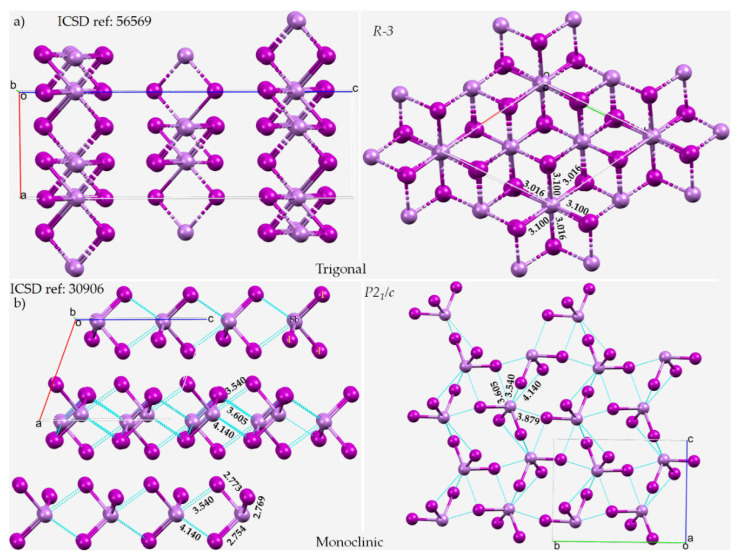
(**a**) The crystal structure of the *R-*$\bar{3}$ trigonal geometry of SbI_3_, showing the layer-like structure in 2D (**left**), and local octahedral coordination environment of the Sb^3+^ ion. (**b**) The crystal structure of the monoclinic geometry of antimony triiodide, showing a 3D network of intermolecular interactions. Selected Sb–I/Sb···I bond distances are shown. The ICSD references are given in each case. Selected bond distances are in Å and degrees, respectively. The intermolecular pnictogen bonding interactions are illustrated as dotted lines in cyan.

By contrast, the SbI_3_ system crystallizes in two space groups, i.e., monoclinic (*P2*_1_*/c*) and trigonal (*R-*$\bar{3}$), and is non-magnetic [139]. The crystal structure of the latter, depicted in Figure 10, shows that it adopts a layered geometry, and that the neighboring layers are linked to each other through a number of Type-IIa halogen-centered I···I interactions (*r*(I···I) = 3.958; ∠Sb–I···I = 164.5°) that are less than twice the vdW radius of I, 4.08 Å. However, in the monoclinic crystal, the isolated SbI_3_ molecules are linked with each other by means of a 3D network of I–Sb···I intermolecular contacts. Both the monoclinic and trigonal structures of SbI_3_ possess wide band gap transition energies [140] (2.217 eV for the monoclinic geometry and 2.211 eV for the trigonal geometry), indicative that they are semiconducting materials. Kang et al. have theoretically determined that SbI_3_ has a strong second harmonic generation effect [133]. Onodera and coworkers have experimentally demonstrated that SbI_3_ is a promising material for use in radiation detectors; it is stable under operating conditions, similar to popular semiconductor detectors CdTe and TlBr [140]. 

**Figure 11:**
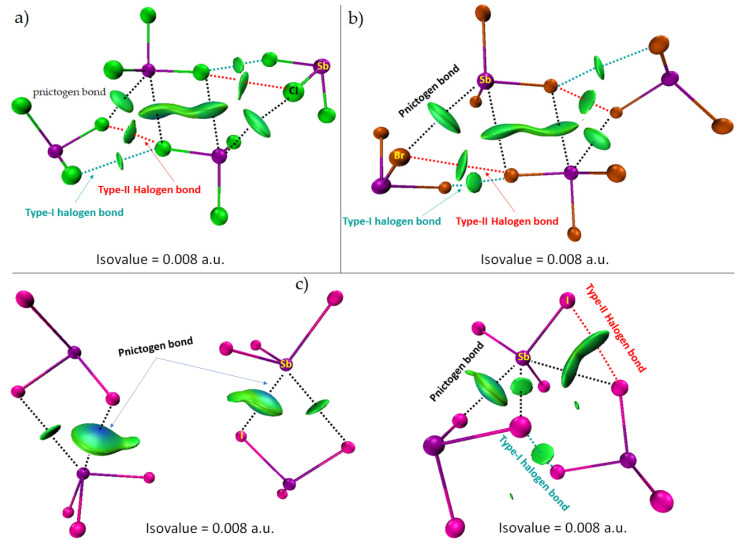
(**a**) IGM-δ*g* based isosurfaces (green and bluish-green volumes between atomic basins) between the molecular entities responsible for the unit-cells of SbX_3_ (X = Cl, Br, I) crystals: (**a**) SbCl_3_ (*Pbnm*); (**b**) SbBr_3_ (*Pbnm*); and (**c**) SbI_3_ (*P2*_1_/*c*). An isovalue of 0.008 a.u. was used on all occasions. The nature of specific intermolecular interactions is shown for each case, and are marked by arrows. Atom type is also shown for all cases. Atoms are shown as small spheres and bonds as lines in atom color, whereas the intermolecular interactions are represented by dotted lines.

The IGM-δ*g* results given in Figure 11 confirm the intermolecular pnictogen bonding interactions that were identified between the interacting molecules in SbX_3_ (X = Cl, Br, I) crystals. As discussed above, we observed that there were circular or dumbbell-shaped green isosurfaces between the Sb and X atomic basins in SbX_3_ (X = Cl, Br). In addition, we also identified a number of potential Type-I and Type-II Sb–X···X halogen bound interactions between the SbX_3_ molecules in the crystal, as marked in Figure 11a,b for SbCl_3_ and SbBr_3_, respectively. In both cases, the pnictogen bonding was stronger than the Type-I and Type-II halogen bonds, as inferred from the size and thickness of the IGM-δ*g* based isosurfaces. The presence of Type-I and -II halogen bonding interactions between the interacting molecules was not obvious in the unit cell of the SbI_3_ crystal. Their presence was rationalized when the unit-cell was expanded. The IGM-δ*g* based isosurface analysis performed on a small portion of the expanded structure of the SbI_3_ crystal is shown in Figure 11c (right), including both Type-I and Type-II I···I halogen bonds in the SbI_3_ crystal. 

### 8.5. The Crystal Structures of [(CH_3_)_3_Sb–Sb(CH_3_)_2_]_2_[(CH_3_SbBr_3_)_2_], (CH_3_)_2_Sb)_2_O and (CH_3_)_2_Sb)_2_S

**Figure 12:**
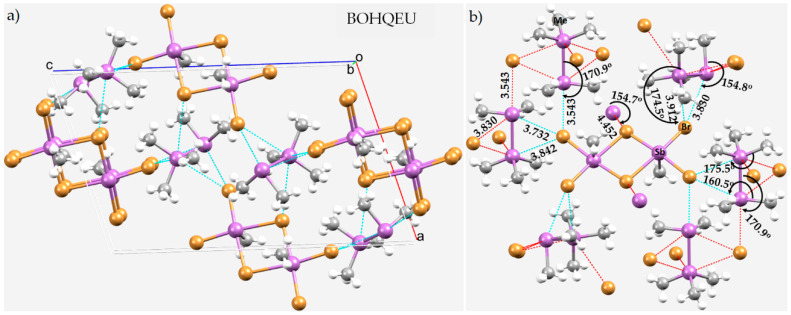
(**a**) The unit-cell of the crystal of (trimethyl-stibino)-dimethyl-stibonium hexabromo-dimethyl-di-antimonate(III), [(CH_3_)_3_Sb–Sb(CH_3_)_2_]_2_[(CH_3_SbBr_3_)_2_], showing Sb···Br interactions. (**b**) Illustration of the nature of Sb-centered non-covalent interactions (dotted lines in cyan) in the crystal. Selected bond lengths and bond angles in Å and degree, respectively are shown. Hanging contacts between atoms are shown as dotted lines in red. The CSD ref. code is shown.

A structure that features prominent charge-assisted Sb···Br contacts, the ionic complex [(CH_3_)_3_Sb–Sb(CH_3_)_2_]_2_[(CH_3_SbBr_3_)_2_], is shown in Figure 12a [141]. Each Br site in [(CH_3_SbBr_3_)_2_]^2–^ is coupled with one, two or three Sb sites of surrounding [(CH_3_)_3_Sb–Sb(CH_3_)_2_]^+^, so forming directional interactions along the C–Sb extensions; see Figure 12b. This means that the Sb site along the Sb–(CH_3_)_2_ bond extensions in [(CH_3_)_3_Sb–Sb(CH_3_)_2_]^+^, form three directional Type-IIa interactions, with Sb···Br bond distances ((H_3_)C–Sb···Br bond angles) of 3.732 (160.5°), 3.549 (170.9°) and 3.830 Å (154.8°). Similarly, the Sb site along the (H_3_C)_3_–Sb bond extensions in the same cation forms three C–Sb···Br intermolecular interactions, with (C)Sb···Br distances of 3.912, 3.842, and 4.452 Å, corresponding to ∠C–Sb···Br of 174.5°, 175.5°, and 154.7°, respectively. 

There are many H···H and H···Br hydrogen bonding interactions in the crystal, with intermolecular bond distances around 2.374 and 2.990 Å, respectively. These are attractive interactions, and each is less than the sum of the van der Waals radii of the respective atomic basins. For instance, the H···H bond distance between the methyl groups is 2.374 Å, which is less than the sum of the vdW radii of two hydrogen atoms, 2.40 Å (*r_vdW_* (H) = 1.20 Å). Similarly, the H···Br distance of 2.990 Å is less than sum of the vdW radii of H and Br atoms, 3.06 Å (*r_vdW_* (H) = 1.20 Å and *r_vdW_* (Br) = 1.86 Å).

**Figure 13:**
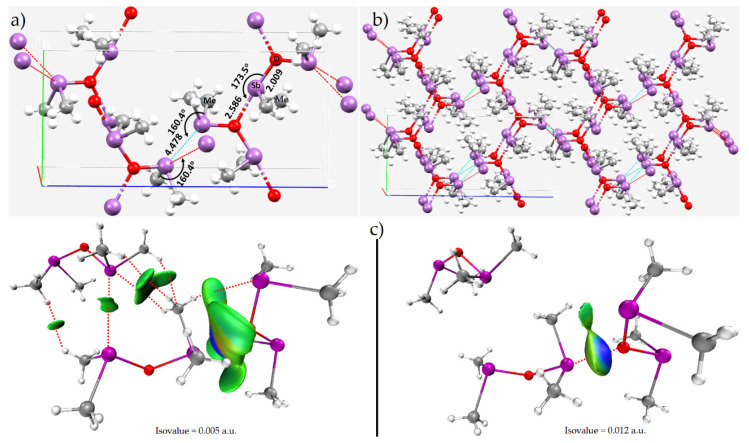
(**a**) Selected intermolecular bonding interactions in the crystal structure of bis(dimethylstibanyl)oxane, ((CH_3_)_2_Sb)_2_O, CSD ref. code MELNAS. (**b**) The 2 × 2 extended structure of the unit-cell of the crystal, showing the Sb···O and Sb···Sb intermolecular contacts. (**c**) The IGM-δ*g* isosurface plots between ((CH_3_)_2_Sb)_2_O entities in the crystal, illustrating the possibility of various intermolecular interactions that appear at low isovalues. Selected bond lengths and bond angles are in Å and degree, respectively. Contacts between atoms are shown as dotted lines in red or cyan. Atom color: Sb—purple; O—red; C—gray; H—white-gray.

Bis(dimethylstibanyl)oxane, ((CH_3_)_2_Sb)_2_O, is an example of an organo-element species that also features pnictogen bonding [142]. As shown in Figure 13a,b, one of the covalently bound terminal Sb sites in ((CH_3_)_2_Sb)_2_O serves as a bifurcated center for donating two pnictogen bonds to a neighboring molecule. The (H_3_)C–Sb···Sb interactions are long and directional: *r*(Sb···Sb) 4.478 Å and ∠(H_3_)C–Sb···Sb = 160.4°. The Sb···Sb interactions are surely characteristics of Type-IIa pnictogen boding; the intermolecular distances are roughly about 10% smaller than the sum of the vdW radius, 4.94 Å, of the two Sb atoms (*r_vdW_*(Sb) = 2.47 Å). They are also markedly longer than the Sb···O pnictogen bond distance between a pair of ((CH_3_)_2_Sb)_2_O entities (*r*(Sb···O) = 2.586 Å and ∠O–Sb···O = 173.5°). The Sb···O distances are longer than the Sb–O covalent bond distances (2.009 Å).

These interactions suggest that Sb in crystalline ((CH_3_)_2_Sb)_2_O is either tetragonal or pentagonal. The authors of [142] suggested that ((CH_3_)_2_Sb)_2_O adopts a *syn-anti* conformation in the solid state with the entities arranged in zigzag chains along because of weak intermolecular Sb···O interactions. We suggest that that these interactions are actually reasonably strong, featuring a significant degree of covalency and may be best described as coordinate bonds. A similar proposition, i.e., that halogen bonds have a significant degree of covalency and are best described as analogous to coordinate bonds rather than analogous to hydrogen bonds, has been advanced [143]. We base our suggestion on the observation that there is a significant accumulation of charge density in the bonding region between the Sb and O atomic basins of a pair of interacting molecules (Figure 13c, right), indicated by blue isosurfaces. This was not so for the Sb···Sb interactions molecules (Figure 13c, left), as the isosurfaces corresponding to these were green, a signature of weakly bound interactions.

Since the Sb–O bond distance in ((CH_3_)_2_Sb)_2_O is 2.009 Å, the mutual penetration between the vdW spheres of Sb and O in within a ((CH_3_)_2_Sb)_2_O entity should be larger than the penetration of these domains with those on neighboring entities. The Sb–O bond is, therefore, a covalent-like bond with some significant ionic characteristics (roughly 38%), which is not unexpected for coordinate bonds [144,145]. Because of its high electronegativity, O in ((CH_3_)_2_Sb)_2_O pulls significant electron density towards the O–Sb bonding region, leaving behind a significantly strong positive region (*σ*-hole) on the surface of the Sb atom along the outermost extension of the O–Sb bond. This *σ*-hole is responsible for formation of a strong O–Sb···O–Sb pnictogen bonding interaction in the crystal; see Figure 13a. The formation of this interaction is the result of two sites of opposite polarity (positive *σ*-hole on Sb in one molecule and negative lone-pair region on O in the neighboring molecule). In addition, there are many O···H and Sb···H interactions between ((CH_3_)_2_Sb)_2_O units in the crystal (not shown), with bond distances (angles) of 2.80–2.90 Å (∠O···H–C = 89 – 100°) and 3.195 Å (∠Sb···H–C = 115°), respectively. This may be inferred from the IGM-δ*g* isosurface plots shown in Figure 13c. 

**Figure 14:**
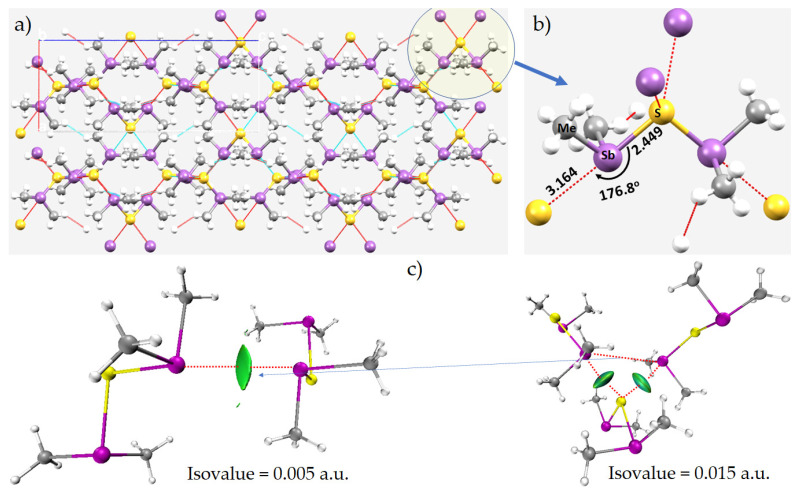
(**a**) The ball-and-stick model of the 2 × 2 supercell structure of crystalline ((CH_3_)_2_Sb)_2_S (CSD ref. code MELNEW). (**b**) The local topology of Sb···S pnictogen bound interactions in the crystal. (**c**) The IGM-δ*g* isosurface plots between the ((CH_3_)_2_Sb)_2_S molecular entities in the crystal, illustrating Sb···Sb and Sb···S interactions. Bond lengths and bond angles are in Å and degree, respectively. Contacts between atoms are shown as dotted lines in red or cyan. Selected atoms are labelled in (**b**).

The crystal structure of the sulfane analog ((CH_3_)_2_Sb)_2_S is also reported (Figure 14a) [142]. The molecules are in an approximately *syn-syn* conformation in the crystal structure. The Sb–S bond length is 2.449 Å, which is significantly smaller than the Sb···S intermolecular distances of 3.164 Å (Figure 14b), due to a pair of interactions that developed when three units of the ((CH_3_)_2_Sb)_2_S came in close proximity in the crystal lattice. Both the Sb···S intermolecular contacts appear along the S–Sb bond extensions with ∠S–Sb···S is = 176.8°, revealing the presence of antimony-centered Type-IIa *σ*-hole interactions. These directional interactions in the crystal are evident in the IGM-δ*g* based bluish-green isosurfaces shown in Figure 14c (right). In addition, there are Sb···Sb long-ranged stibium bonds in the crystal, with *r*(Sb···Sb) = 4.208 Å and ∠S–Sb···Sb =134.8°. These are Type-Ib pnictogen bonds, and show up in the IGM-δ*g* based green isosurfaces shown in Figure 14c (left). There are many H···S hydrogen bonds in the extended crystal lattice (not shown). 

### 8.6. The Crystal Structure of Catena-(tris(μ_2_-1,4-dioxane-O,O’)-bis(trichloro-antimony(III))

In the organic–inorganic hybrid complex formed between SbCl_3_ and 1,4-dioxane, the Sb^3+^ ion is in a pseudo-octahedral environment, coordinated to three chloride ligands and interacting with the lone-pair electrons on three O-sites in three surrounding 1,4-dioxane moieties [146,147].

**Figure 15:**
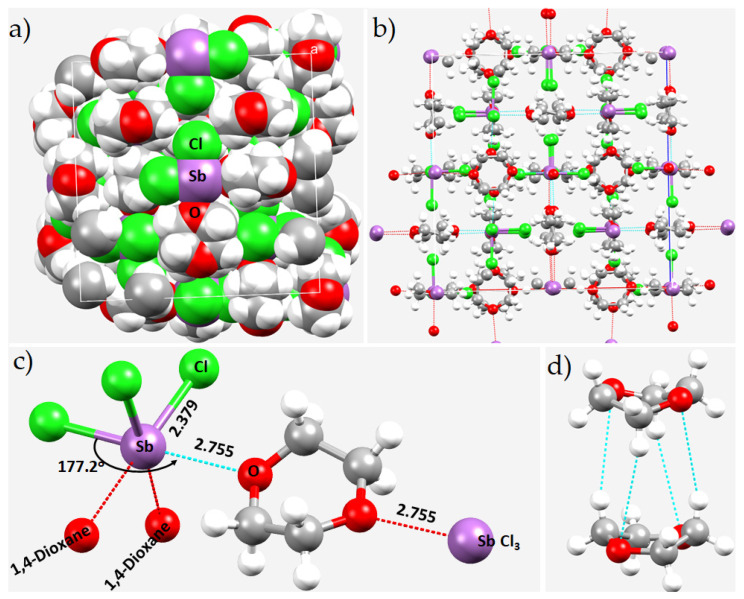
(**a**) The space-filling and (**b**) ball-and-stick models of the coordination of SbCl_3_ with 1,4-dioxane as observed in the crystal of catena-(tris(μ_2_-1,4-dioxane-O,O’)-bis(trichloro-antimony(III)), CSD ref. code BEKHUV01). (**c**) Illustration of local Sb···O links in the crystal. (**d**) Hydrogen bonding topologies between 1,4-dioxane moieties in the crystal. Selected bond lengths and bond angles are in Å and degree, respectively. Hanging contacts are shown as dotted lines in red. Atoms are shown as small spheres and bonds as lines in atom color, whereas the intermolecular interactions are represented by dotted lines in cyan.

The space-filling and ball-and-stick models of the compound are shown in Figure 15a,b. The first shows that there is an overlapping of Sb and O sites in the crystal, causing the formation of three equivalent Cl–Sb···O interactions (Figure 15c). The Sb···O distance, *r*(Sb···O), is 2.755 Å and the angle of interaction, ∠Cl–Sb···O, is 177.2°. These Type-IIa stibium bonds are markedly longer than the three formal Sb–Cl coordinate bonds (2.379 Å) of SbCl_3_. The Sb···O interactions can be viewed as the result of attraction between the regions of positive potential along the Cl–Sb bond extensions and the negative potential on the surface of O atoms in 1,4-dioxane. One may also expect that they have some characters expected of coordinate bonds, and are largely coulombic interactions. 

In addition, there are also Cl···Cl halogen bonds and H···O and H···Cl hydrogen bonds between the interacting molecules in the crystal. The first developed between the Cl sites on the Sb–Cl bond extensions, with *r*(Cl···Cl) bond distances of 3.912 Å and angle of interaction, ∠Sb–Cl···Cl, of 172.85°. Although these interactions are directional and have a Type-IIa topology of bonding, they are not actually Type-IIa interactions (∠Sb–Cl···Cl = 172.85°; and ∠Cl–Cl···Cl = 172.85°), since the Sb–Cl···Cl–Sb interactions are formed between regions of nearly identical electrostatic potentials centered on the interacting Cl atoms. They may therefore be best characterized as Type-III halogen bound interactions [22]. On the other hand, the C–H···Cl hydrogen bonds are formed between the negative sites on the Cl atoms bonds around the Sb–Cl bonds and the nearest H sites on 1,4-dioxane, with *r*(H···Cl) varies in the range 2.9–3.3 Å. Each Cl site in SbCl_3_ serves as an acceptor of six H···Cl hydrogen bonds, with bond distances of 2.928, 3.023, 3.106, 3.145, 3.148 and 3.198 Å. The C–H···O hydrogen bonds in the crystal are formed between the 1,4-dioxane moieties, the majority of them with a bond distance of 3.315 Å and an interaction angle of 161.1°. There are four such bonds between a pair of two 1,4-dioxane molecules, locally forming a sandwich type 1,4-dioxane dimer (Figure 15d). Most of the hydrogen bonds are weak, and they originate from attraction between the positive site on H and the negative site(s) on Cl or O, making a local contribution to the overall stability of the crystal. 

### 8.7. The Crystal Structure of [Co(trien)(NSC)_2_][Sb_2_(tart)(Htart)]

**Figure 16:**
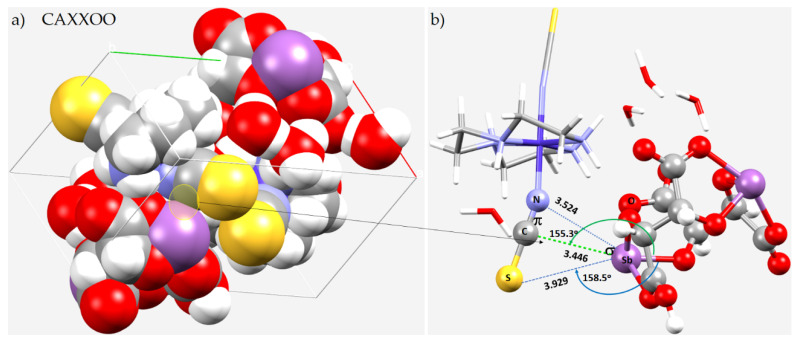
(**a**) The space-filling model of crystalline [Co(trien)(NSC)_2_][Sb_2_(tart)(Htart)], CSD ref. code CAXXOO. (**b**) The ball-and-stick model of the same structure, showing atom type and highlighting the possibility of π-centered non-covalent interactions. Bond lengths and bond angles are in Å and degree, respectively. Atoms are shown as small spheres and bonds as lines in atom color, whereas the intermolecular interactions are represented by dotted lines.

Kushi and co-workers [148] reported the structure of the diastereomeric salt formed between *trans*-[Co(trien)(NSC)_2_]^+^ (trien = triethylenetetramine) and [Sb_2_(tart)(Htart)]^–^ (tart = tartrate, C_4_H_2_O_6_^2–^). It features each Sb^3+^ ion coordinated to four O atoms of tartrate in a distorted seesaw coordinated environment. Sb is engaged in an attractive engagement with a *π*-cloud around the C=N bond of the coordinated isothiocyanato anion (Figure 16a,b). The N=C and C=S bonds in the metal coordinate SCN^–^ anion form two interactions with an Sb^3+^ ion of the cation, Sb(σ)···π(SCN). These are probably weak, charge assisted interactions, corresponding to *r*(Sb···π(midpoint of N=C)) = 3.437 Å and *r*(Sb···π(midpoint of C=S) = 3.607 Å). The sum of the vdW radii of Sb and S atomic basins is 4.36 Å, considerably longer than the distance between Sb and S (3.929 Å; see Figure 16b). The interaction is also directional, yet Type-IIa as ∠O–Sb···S = 158.5°. This contact between Sb and S in the crystal is not a pnictogen bond, but rather, the result of charge assisted π-hole centered tetrel bonding, since *trans*-[Co(trien)(NSC)_2_]^+^ carries a formal charge of +1. Similarly, Sb···π(midpoint of N=C) is a π-hole centered tetrel bond, yet Sb is closer to C than to N. These two interactions, together with the four Sb–O bonds to tartrate, make the coordination environment of Sb^3+^ pseudo-octahedral in this salt (Figure 16b). Apparently, the packing in the crystal is not only driven by chalcogen and tetrel bonding interactions, but also by several other non-covalent interactions formed between the interacting species (not shown). This example implies that interactions that appear to be the result of pnictogen bonding may actually correspond to something else if assigned correctly. 

### 8.8. The Crystal of [SbCl_2_imR_2_R’_2_][OTf]

**Figure 17:**
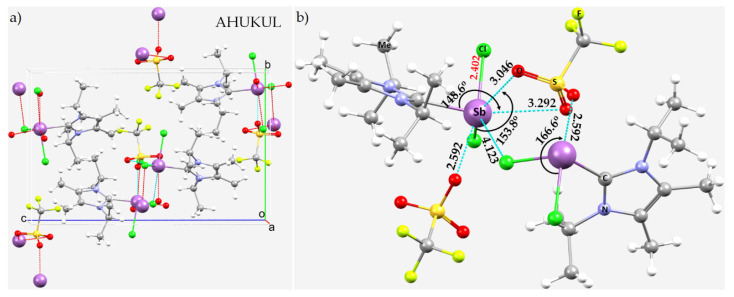
(**a**) The unit-cell crystal structure of [SbCl_2_imR_2_R’_2_][OTf]. (**b**) The nature of coordination environment around the Sb center is highlighted. The hanging contacts represented by dotted lines in red are Sb···O pnictogen bonding interactions. The CSD reference code is shown, and selected bond lengths and bond angles are in Å and degrees, respectively. Atoms are shown as small spheres and bonds as lines in atom color, whereas the intermolecular interactions are represented by dotted lines in cyan/red.

Henne et al. reported a series of pnictogen complexes featuring a Pn–C bond in the C2 position of a sterically hindered imidazole (imR_2_R’_2_ = 1,3-dipropyl-2,5-dimethyl imidazole) [149]. One of these, [SbCl_2_imR_2_R’_2_][OTf] (OTf = triflate), is shown in Figure 17a. As discussed above for other systems, the solid-state structure arises from several charge-assisted intermolecular interactions, as Sb is entirely positive. The three Sb···O contacts shown in Figure 17b are a result of coulombic attraction between it and the entirely negative O sites on two neighboring OTf anions. Two of these interactions are significantly longer than the other one (2.592, 3.046 and 3.292 Å), with corresponding ∠C–Sb···O angles of 166.6°, 153.8°, and 148.6°, respectively. The shorter of these interactions has a comparable bond length to the two Sb–Cl bonds (2.402 and 2.375 Å); one may recognize this as having the significant character of any coordinate bond. The other two display characteristics of Type-II non-linear pnictogen bonds. 

We also identified a long Sb···Cl bond between neighboring cations with r(Sb···Cl) = 4.123 Å and an ∠Cl–Sb···Cl = 129.4° (Type-IIb). This is also a weak, non-linear pnictogen bond, given that it is marginally shorter than the sum of the vdW radii of the Sb and Cl atomic basins, 4.29 Å. The Sb center in [SbCl_2_imR_2_R’_2_]^+^ is therefore pseudo seven coordinate. 

### 8.9. The Crystal Structures of Phosphoryl Isothiocyanate

**Figure 18:**
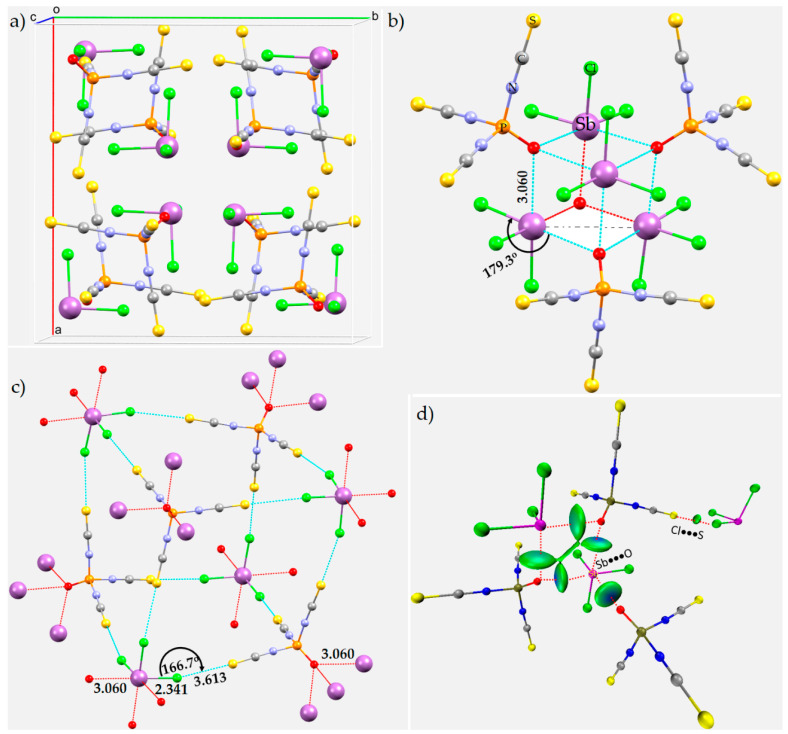
(**a**) The unit-cell of crystalline [SbCl_3_·OP(NCS)_3_]_4_ (ICSD ref. code 80097). (**b**) The nature of the pseudo-octahedral coordination environment around the Sb center. (**c**) The Cl···S intermolecular interactions responsible for the pseudo 3D geometry of the crystalline material. (**d**) The IGM-δ*g* based isosurface topologies (isovalue = 0.008 a.u.) between OP(NCS)_3_ and SbCl_3_. Bond distances and bond angles are in Å and degree, respectively. Hanging contacts in (**b**,**c**) in red represent coordinate bonds/intermolecular interaction. Atoms are shown as small spheres and bonds as lines in atom color, whereas the intermolecular interactions are represented by dotted lines.

Phosphoryl isothiocyanate, OP(NCS)_3_ can form many types of crystal structures [150]. An example is [SbCl_3_·OP(NCS)_3_]_4_, which crystallizes in the cubic space group *I*$\bar{4}$*3m*, shown in Figure 18a [150]. The three chloride ligands of SbCl_3_ are arranged in a primarily trigonal pyramidal geometry around Sb^3+^, which also interacts with three O sites of three neighboring OP(NCS)_3_ units.

As shown in Figure 18b, there are three links between the Sb center in SbCl_3_ and the three O sites of OP(NCS)_3_ moieties. These are a result of coulombic attractions between the positive potential on Sb and the negative potential associated with the lone-pair electrons on O. They are all equivalent and are much longer (3.060 Å) than the formal Sb–Cl bonds (2.341 Å). Because the Sb center interacts with six atomic basins, this creates a pseudo-octahedral around it. Therefore, the complex system exhibits a tetranuclear “cage” structure with an [Sb_4_O_4_] core, in which four corners of the core form a regular cube of edge length 3.060 Å that are occupied by four Sb^3+^ ions and the remaining four are occupied by O sites. The [Sb_4_O_4_] core consists of *μ*_3_-bridging oxygen and six coordinated Sb^3+^ ion such that each O in OP(NCS)_3_ acts as a trifurcated center to accept electrophilic attacks. All four lone pairs of the four Sb atoms suggested in ref. [150] are located in the tetranuclear closed “cage” structure of the [Sb_4_O_4_] core. Because the Sb···O intermolecular interactions are substantially longer than the Sb–Cl bonds, and appear along the Cl–Sb extensions (∠Cl–Sb···O = 179.3°), we characterize them as Type-IIa stibium bonds. 

Our further analysis suggested that Cl, along the Sb–Cl bond extension, also engages attractively with the S atom in OP(NCS)_3_. Each SbCl_3_ unit in the crystal donates three chlorine-centered *σ*-holes to three S, forming three equivalent Cl···S halogen bonds (*r*(Cl···S) = 3.613 Å and ∠Sb–Cl···S = 166.7°). These halogen-centered *σ*-hole interactions leads to the emergence of macrocyclic cage-like pseudo 3D structures as shown in Figure 18c. 

The antimony atoms form a distorted tetrahedron with an Sb···Sb edge of 4.186(1) Å, which is much less than the sum of the vdW radii of two Sb atoms (4.94 Å). Because of this, a weak specific interaction between Sb ions was postulated [150]. Other than this, we also expect that the crystal should contain weak (CN)π···Cl interactions. These postulated intermolecular interactions that are responsible for the stability of the [SbCl_3_·OP(NCS)_3_]_4_ crystal are elucidated by our IGM-δ*g* analysis. The results are summarized in Figure 18d for a small portion of the crystal, consisting of three SbCl_3_ units and three OP(NCS)_3_ units. The IGM-δ*g* results show that the Sb···Sb pnictogen bonds are very weak, and appear as a thin flat isosurface colored green. The Cl···S interaction is a localized interaction and the isosurface volume between Cl and S is tiny. By contrast, the isosurface volumes between the Sb and O atomic basins are thick and circular, and bluish-green, which indicate strongly bound stibium bonds. 

**Figure 19:**
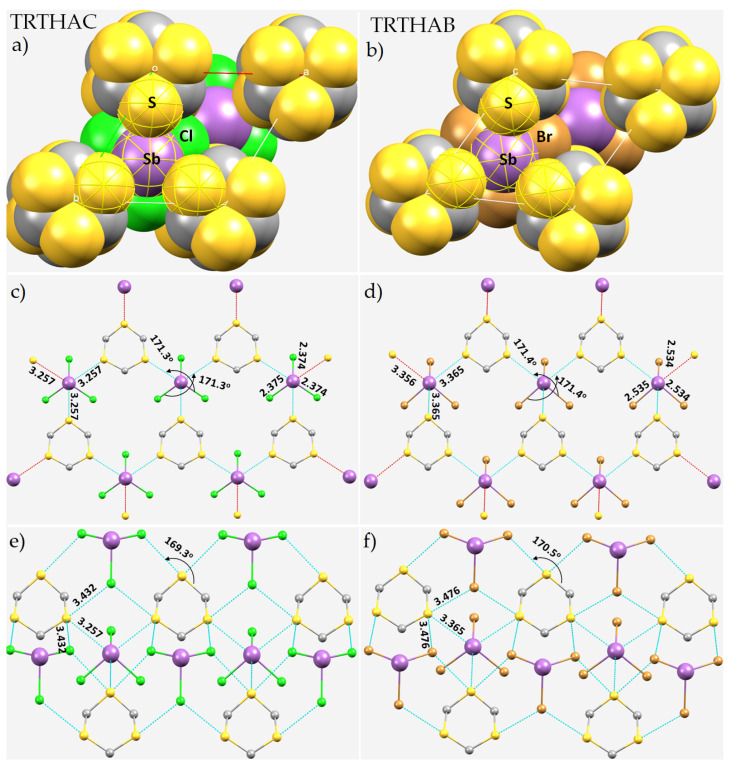
Space-filling models showing the molecular packing in the crystals of (**a**) SbCl_3_·(CH_2_S)_3_ (CSD ref. code in capital letters) and (**b**) SbBr_3_·(CH_2_S)_3_ (CSD ref. code TRTHAB). Illustration of the exceptionally long Sb···S intermolecular distances and their angular features is shown in (**c**,**d**), respectively. Included in (**e**,**f**) are the *μ*_3_-bridges of covalently bound S, leading to the emergence of Type-IIa chalcogen bonding in SbCl_3_·(CH_2_S)_3_ and SbBr_3_·(CH_2_S)_3_ crystals, respectively. Atom types are labeled in each case. Atoms are shown as small spheres and bonds as lines in atom color, whereas the intermolecular interactions are represented by dotted lines in cyan. Bond distances and bond angles are in Å and degree, respectively.

Similar pnictogen bonding topologies were found in a crystal of 1,3,5-Trithiane antimony tribromide, SbX_3_·(CH_2_S)_3_ (X = Cl, Br) [151]. The packing between SbX_3_ and (CH_2_S)_3_ is illustrated in Figure 19a,b for SbCl_3_·(CH_2_S)_3_ and SbBr_3_·(CH_2_S)_3_, respectively. The H atom positions are not provided in the CSD deposition, and thus, are missing in Figure 19. The intermolecular interactions between the interacting units in the crystal are shown in Figure 19c,d, respectively. Because of the absence of H atom positions in CH_2_S in the crystal, the probable role of H cannot be determined. H is expected to play a role in the packing by the formation of several non-covalent interactions. One such prominent interaction in SbBr_3_·(CH_2_S)_3_ could be the (HC)H···Br/(C)H···Br hydrogen bonds, for example.

As found in other structures, see above, the Sb center in SbX_3_·(CH_2_S)_3_ (X = Cl, Br) is pseudo-octahedral. In SbCl_3_·(CH_2_S)_3_, the Sb–Cl distances are 2.374 Å, and the Sb···S distances are much longer, i.e., 3.257 Å. Similarly, in SbBr_3_·(CH_2_S)_3_, the Sb–Br distances are 2.534 Å, while the Sb···S distances are, 3.365 Å. The presence of the exceptionally Sb···S long bonds signifies that they are not coordinate bonds, but Type-IIa stibium bonds, and that the SbX_3_ units are locally trigonal pyramidal. We note further that there are many S···Cl and S···Br chalcogen bonds in the crystal, and each S site is involved in *μ*_3_-bridges; two of them are equivalent S···X chalcogen bonds (*r*(S···Cl)/*r*(S···Br) = 3.432/3.476 Å; ∠C–S···Cl/∠C–S···Br = 169.3°/170.5°) and the other is the Sb···S pnictogen bond (see above). The chalcogen bonds arise from the two σ-holes on S along the C–S bond extensions (not shown).

### 8.10. The Crystal Structure of (CN_4_H_7_)SbC_2_O_4_F_2_(H_2_O)_0.5_

**Figure 20:**
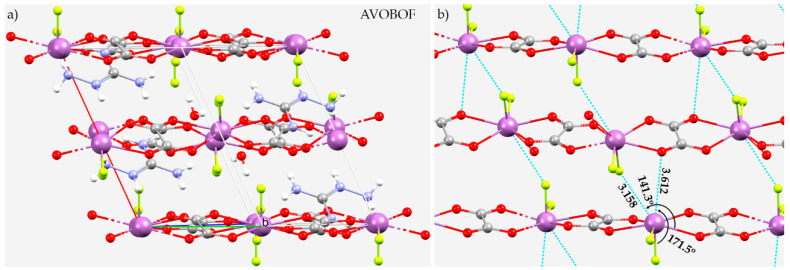
(**a**) The unit-cell of the crystal structure of (CN_4_H_7_)SbC_2_O_4_F_2_(H_2_O)_0.5_. Shown in (**b**) is the local coordination environment around the Sb center in the crystal. Selected bond lengths and bond angles are in Å and degree, respectively. The CSD ref. code in capital letters is marked. Atoms are shown as small spheres and bonds as lines in atom color, whereas the intermolecular interactions are represented by dotted lines in cyan. Atom color: Sb—purple; O—red; F—green; C—gray; H—white-gray; N—blue. The [CN_4_H_7_]^+^ and H_2_O moieties in (**b**) are removed for clarity.

Chen and co-workers reported an organic–inorganic hybrid birefringent material, i.e., (CN_4_H_7_)SbC_2_O_4_F_2_(H_2_O)_0.5_, comprising π-conjugated organic groups [CN_4_H_7_]^+^ and linear chains of [SbF_2_]^+^ units bridged by oxalate anions, [SbF_2_(C_2_O_4_)]^−^_n_, with amino-guanidinium cations intercalated between the chains (Figure 20a) [152]. The compound exhibits a large birefringence (Δ*n* = 0.126 @ 546 nm) that is almost equal to that of the well-known birefringent material, α-BaB_2_O_4_. Sb is bound to four O and two F donors, forming a distorted pentagonal pyramidal structure. There is therefore significant space around the coordination sphere of Sb, opposite the F–Sb bonds, for which the Sb center is able to accommodate interactions with negative sites, just as we observed. The positive site on Sb is involved in F–Sb···O (oxalate) and F–Sb···F pnictogen bonding interactions (Figure 20b) with bond distances of 3.612 and 3.158 Å, and with corresponding angles of interactions of 171.5° and 141.3°, respectively. These intermolecular distances are less than the sum of the vdW radii of the respective bound atomic basins, and the intermolecular interactions are directional. 

Each chain formed by the repetition of the [SbF_2_C_2_O_4_]^–^ units is linked to a neighboring chain by F–Sb···O (oxalate) and F–Sb···F pnictogen bound interactions (Figure 20b). The π-conjugated [CN_4_H_7_]^+^ and the H_2_O moieties act as spacers between the SbC_2_O_4_F_2_ chains. They are linked to each other through N–H···O (oxalate), N–H···F(Sb), (H_2_O)H···O(oxalate), (H_2_O)H···F(Sb), –NH···O(H_2_O) and N–N···O contacts (not shown). While the first five are genuine hydrogen bonds, the last are potentially charge-assisted pnictogen bonds. Because *r*(N···O) distances occur in the 2.982–3.200 Å range and ∠N–N···O = 84.6° and 140°, these can be regarded as Type-I pnictogen bound contacts not shown. Apart from these interactions, H_2_O molecules are linked with the Sb sites through Sb···O pnictogen bonds (*r*(Sb···O) = 3.082 Å and ∠F–Sb···O = 148.6°). 

### 8.11. Stibium Bonds Formed with Arene Moieties and Other Cyclic and Non-Cyclic Systems 

**Figure 21:**
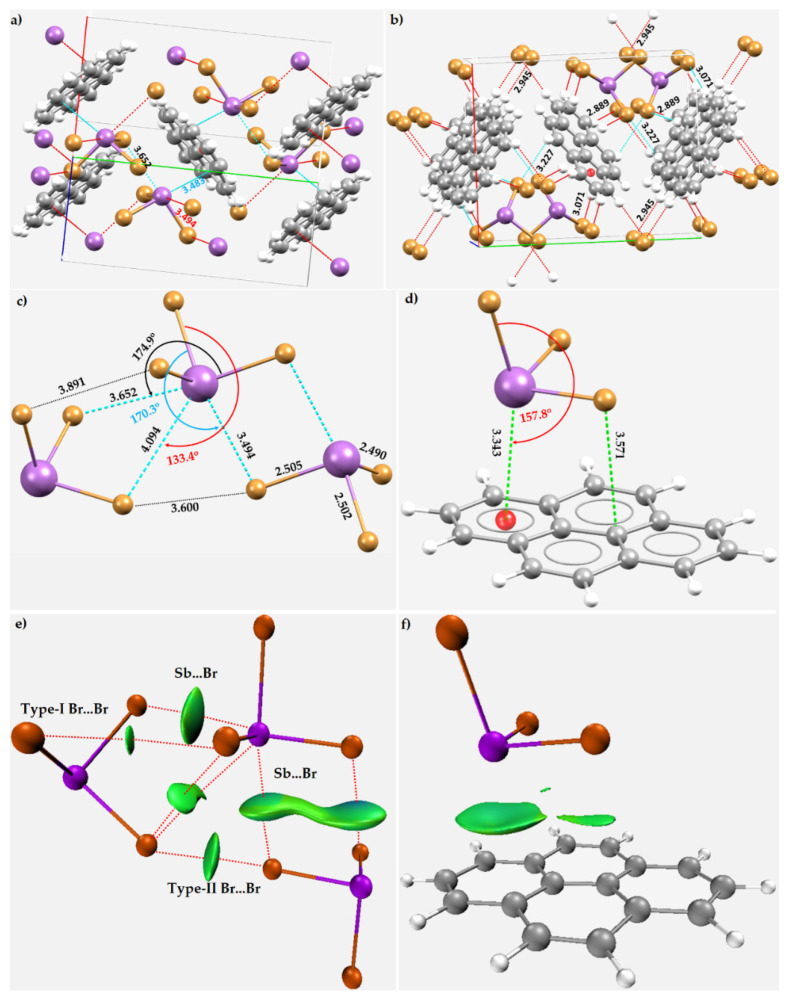
The topology of (**a**) Sb···Br and Sb···π pnictogen bonding and (**b**) hydrogen bonding in the unit-cell of the crystal of SbBr_3_ (CSD ref. code PYRABR). (**b**) The nature of Br···Br halogen-halogen bonding and Sb···Br pnictogen bonding between the SbBr_3_ units in the crystal. (**c**) The nature of Sb···π pnictogen bonding between pyrene and SbBr_3_ in the crystal. (**e**,**f**) The IGM-δ*g* based isosurface topologies between the molecular entities shown in (**c**) and (**d**), respectively. Selected bond distances and bond angles are in Å and degree, respectively. Atoms are shown as small spheres and bonds as lines in atom color, whereas the intermolecular interactions are represented by dotted lines. Atom color: Sb—purple; Br—dark-red; C—gray; H—white-gray.

Covalently bound Sb can form pnictogen bonds with π-density rich regions. For example, Bombieri and co-workers [153] reported such a case, i.e., the 2:1 complex between SbBr_3_ and pyrene (Figure 21a). The figure illustrates the prominence of pnictogen bonds between interacting SbBr_3_ units, as well as between SbBr_3_ and pyrene. Sb^3+^ in each SbBr_3_ unit simultaneously forms three Br–Sb···Br pnictogen bonds with the Br sites in neighboring SbBr_3_ units, which act as pnictogen bond acceptors. At the same time, the Sb forms a pnictogen bond with the π-density on the aromatic rings of pyrene which also act as pnictogen bond acceptors. Most of the Br–Sb···Br and Br–Sb···π(C) interactions are directional, with different ∠Br–Sb···Br values of between 170° and 175° (Figure 21c), and ∠Br–Sb···π(C_6_) = 157.8° (Figure 21d).

The lateral negative sides around Br in SbBr_3_ are also involved in an extensive number of C–H···Br hydrogen bonds. There are many such intermolecular non-covalent interactions between SbBr_3_ and pyrene, with Br···H distances varying between 2.85 and 3.20 Å. They display a Type-IIa directional feature, with the angle of interaction, ∠C–H···Br in the 150.0–170.0° range, as shown in Figure 21b. There are also Type-I hydrogen bonds; they are longer, with *r*(H···Br) ~ 3.0–3.3 Å, and ∠C–H···Br in the 90.0–150° range.

The crystal also features a significant number of Sb–Br···Br(Sb) contacts between the SbBr_3_ units, between 3.60 and 3.90 Å in length. The bond distances for Type-I Sb–Br···Br(Sb) contacts occurring between the SbBr_3_ units are close to 3.89 Å, with bond angles lying between 90° and 120° (Figure 21c). The ability of Br in SbBr_3_ to engage in non-covalent interactions is remarkable. It is involved either in (i) two Type-I and one Type-II Br···Br halogen bound interactions (∠Sb–Br···Br = 160.3°); or (ii) in three Type-I and one Type-II Br···Br contacts; or (iii) in one hydrogen bond, one pnictogen bond, one Type-II and two Type-I Br···Br contacts; (iv) or in a set of one Sb–Br···Br pnictogen bond and two Br···H hydrogen bond(s); (v) or in a set of two halogen bonds, one pnictogen bond, and one hydrogen bond; (vi) or in three halogen bonds and one hydrogen bond; (vi) or in a set of four halogen bonds and one hydrogen bond; (vii) or in a set of one pnictogen bond, two hydrogen bonds, and one Br···π(pyrene ring) interactions.

In addition, there are also slipped-parallel π···π staking interactions between the aromatic rings of pyrene. This complicated bonding topology emerges from the variable orientation of the SbBr_3_ molecule in the crystal, which plays a crucial role in packing with the pyrene entity. While all types bonding interactions responsible for the crystal are not explicitly discussed, in Figure 21e,f, we present the IGM-δ*g* based isosurface results calculated between three SbBr_3_ units, and those between SbBr_3_ and pyrene, the geometries of which were extracted from the crystal. As discussed above, our IGM-δ*g* analysis provides evidence of Type-I and Type-II halogen bonding between the SbBr_3_ units—described by circular thin green volumes (Figure 21e); Sb···Br pnictogen bonds—described by bluish-green circular or dumbbell-shaped volumes (Figure 21e); and Sb···π pnictogen bonds—described by a cone-like volume (Figure 21f). 

A few systems dominated by Sb···π pnictogen bonds are included in Figure 1, including, as examples, (C_24_H_24_)(SbCl_3_)_2_ (C_24_H_24_ = [2.2.2]paracyclophane [110] (Figure 1c); biphenyl bis(antimony trichloride) [111] (Figure 1d); (benzene)(SbCl_3_)_2_ [115] (Figure 1h); pyrene bis(trichloro-antimony) (Figure 1i) [116]; [2.2.2.2]-paracyclophane tribromo-antimony (Figure 1j) [117]; trichloro-antimony toluene (Figure 1k) [118]; and tetrakis(trichloro-antimony) 1,2-diphenylethyne (Figure 1l) [119]. In all cases, the intermolecular distances are less than 3.5 Å, and not unexpected for π-centered intermolecular interactions.

**Figure 22:**
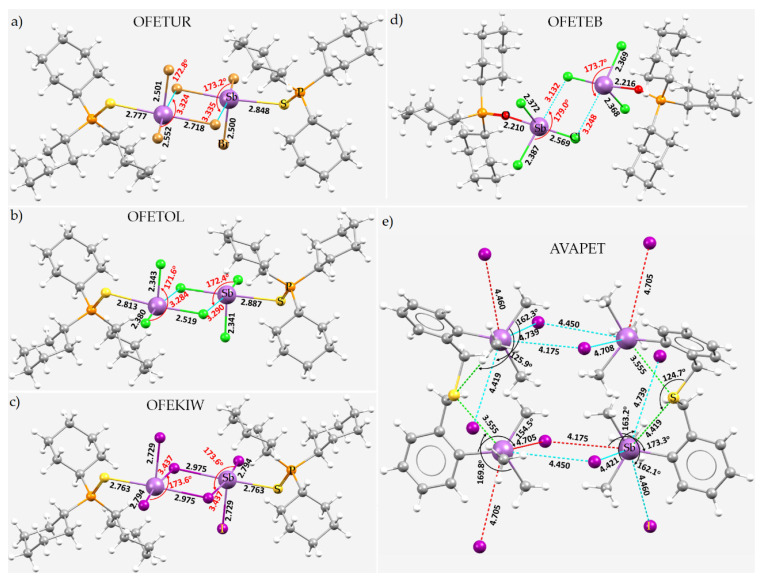
(**a**) The nature of the coordination environment around the Sb ion in the crystals of (**a**) (Cy_3_PS)SbBr_3_, (**b**) (Cy_3_PS)SbCl_3_, (**c**) (Cy_3_PS)SbI_3_, (**d**) (Cy_3_PO)SbCl_3_, and (**e**) [S(CH_2_-2-C_6_H_4_SbMe_3_)_2_]I_2_. Selected bond lengths and bond angled in Å and degree, respectively. Atoms are shown as small spheres and bonds as lines in atom color, whereas the intermolecular interactions are represented by dotted lines. Atom color: Sb—purple; O—red; Br—dark-red; C—gray; H—white-gray; P—orange; S—yellow; Cl—green. CSD ref. codes are shown.

In any case, Karlee et al. reported tricyclohexylphosphine sulfide antimony trihalide crystals with generic formulae (Cy_3_PO)SbX_3_ (X = F, Cl, or Br), (Cy_3_PO)_2_SbX_3_ (X = F, Cl, or Br), and (Cy_3_PS)SbX_3_ (X = Cl, Br, or I) [154]. (Cy_3_PO)SbX_3_ (X = F, Cl) crystallizes as dimers through symmetry-related intermolecular Sb···X interactions. A similar type of dimeric structure was observed for (Cy_3_PS)SbX_3_ (X = Cl, Br). By contrast, the solid-state structure of (Cy_3_PO)_2_SbCl_3_ was consistent with the structures of *bis*-phosphine oxide complexes of Sb^3+^ that have a square pyramidal Sb center and cis-configured OPCy_3_ ligands. The crystal structures of (Cy_3_PS)SbBr_3_, (Cy_3_PS)SbCl_3_, and (Cy_3_PS)SbI_3_ are shown in Figure 22a, Figure 22b and Figure 22c, respectively.

In all these phosphine chalcogenide complexes, the Sb–X bond distances are significantly distorted from what is expected of a trigonal pyramidal SbX_3_ molecular entity. The three Sb–X bonds of SbX_3_ in its complex with Cy_3_PS are not equivalent, and each of them is significantly shorter than the Sb–S bond. The Sb···X bonds responsible for the (Cy_3_PS)SbX_3_ (X = Cl, Br, I) dimers are significantly longer than ordinary coordinate bonds, and are directional. They are inequivalent in (Cy_3_PS)SbBr_3_ (3.335 and 3.324 Å) and (Cy_3_PS)SbCl_3_ (3.290 and 3.284 Å), but equivalent in (Cy_3_PS)SbI_3_ (3.437 Å). The Sb···X interactions are quasi-linear (∠X–Sb···X (X = Cl, Br, I) between 171.6° and 173.6°), indicating that directionality plays a role in stabilizing these interactions. A very similar coordination environment around the Sb cation is also found in (Cy_3_PO)SbCl_3_, as shown in Figure 22d. In this case, the inequivalent Sb···Cl bond distances are 3.132 and 3.248 Å with corresponding ∠Cl–Sb···Cl values of 173.7° and 179.0°, respectively. Other than X–Sb···X pnictogen bonding, the H-sites of the Cy moieties are linked with the nearest X sites of the coordinated SbX_3_, forming a number of weak H···Br hydrogen bonds, with bond distance in the range, 3.00–3.50 Å (not shown).

**Figure 23:**
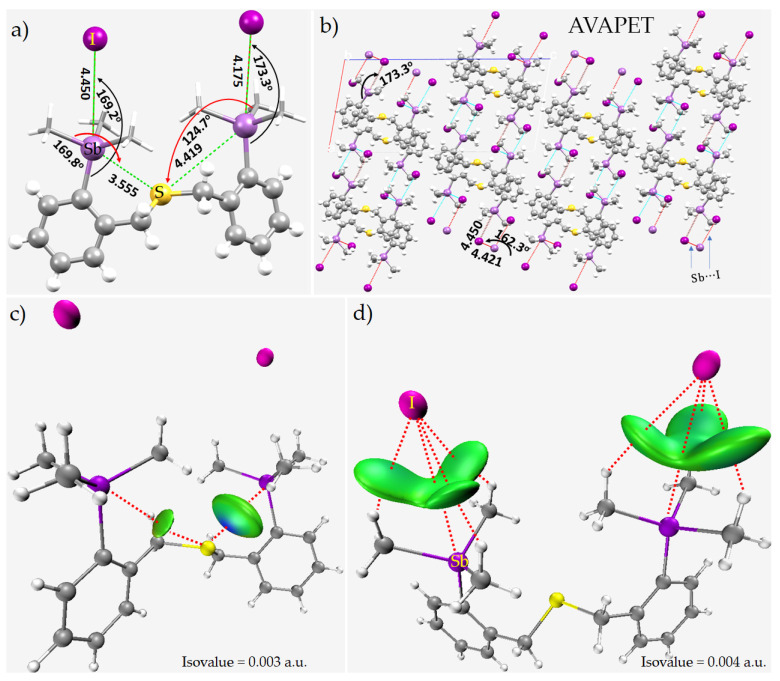
(**a**) Charge-assisted C–Sb···I pnictogen bonding in [S(CH_2_-2-C_6_H_4_SbMe_3_)_2_]I_2_. (**b**) The 2 × 2 × 2 unit-cell structure of the crystal, showing the C–Sb···I pnictogen bond networks in the crystal. (**c**) and (**d**) Illustration of the IGM-δ*g* based isosurface topologies of the C–Sb···S and C–Sb···I pnictogen bonds in (**a**). The detailed nature of pnictogen bonding around the Sb cation may be inferred from Figure 22e (see above). Selected bond distances and angles in Å and degrees, respectively. Atom type and CSD ref. codes in capital letters are shown. Atoms are shown as small spheres and bonds as lines in atom color, whereas the intermolecular interactions are represented by dotted lines.

The structure of [S(CH_2_-2-C_6_H_4_SbMe_3_)_2_]I_2_, resulting from pnictogen bonding and extensive other primary and secondary interactions, is shown in Figure 22e and Figure 23 [155]. A representation of the ionic species, [S(CH_2_-2-C_6_H_4_SbMe_3_)_2_]^2+^·2I^−^, is shown in Figure 23a, and the expanded crystal system is shown in Figure 23b. Since the pnictogen bonding is between (formally) ionic species, it is charge assisted. 

While appreciating that [S(CH_2_-2-C_6_H_4_SbMe_3_)_2_]I_2_ is an ionic species, for convenience, we shall refer to it as a “molecular unit”. The detailed topology of antimony-centered pnictogen bonding cannot be inferred from the molecular unit shown in Figure 23a, and is best appreciated from Figure 22e. There are two different C–Sb···I links and two different C–Sb···S links, two intermolecular, and two intramolecular. Except for the C–Sb···S bond with an intramolecular distance of 3.555 Å, the other three are significantly longer. The C–Sb···I links have the characteristics of Type-II pnictogen bonds, whereas one of the two C–Sb···S bonds, at an intramolecular distance of 4.419 Å, is a characteristic of Type-I. Each of the contacts is shorter than the sum of the vdW radii of the respective atomic basins, 4.51 Å. Similarly, the intramolecular distance of one of the two C–Sb···S bonds (*r*(Sb···S) = 3.555 Å) is shorter than the sum of the vdW radii of S and Sb, 4.36 Å, while the other is slightly longer. These observations indicate the formation of two intramolecular hypervalent C–Sb···S pnictogen bonds. The nature of the intermolecular interactions is revealed by inspecting the molecular ordering in the expanded crystal, a part of which is shown in Figure 22e. 

Each of the two Sb centers of the cation is formally four-coordinate. Since each Sb center has four positive *σ*-holes along the four C–Sb bond extensions, they are in coulombic engagement with four nearest iodide anions. The resulting interactions, i.e., C–Sb···I, are all long-ranged and directional. We also note that the (Me)H···I hydrogen bonds are key driving forces causing the occurrence of these charge-assisted pnictogen bonds in the crystal system (*r*(H···I) = 3.0–3.5 Å). To confirm these observations, we performed an IGM-δ*g* based isosurface analysis, using the geometry of the ion-pair shown in Figure 23a. The results are summarized in Figure 23c,d. The first suggests that the IGM-δ*g* isosurface volumes in green and bluish-green representing the long and short interactions between Sb and S within the cation are weak and medium strength interactions, respectively. Similarly, the broad isosurface volumes in green appearing between I and H atomic basins in Figure 23d suggest that the (Me)H···I hydrogen bonds are the primary interactions that lead to the formation of C–Sb···I pnictogen bonds in the crystal.

**Figure 24:**
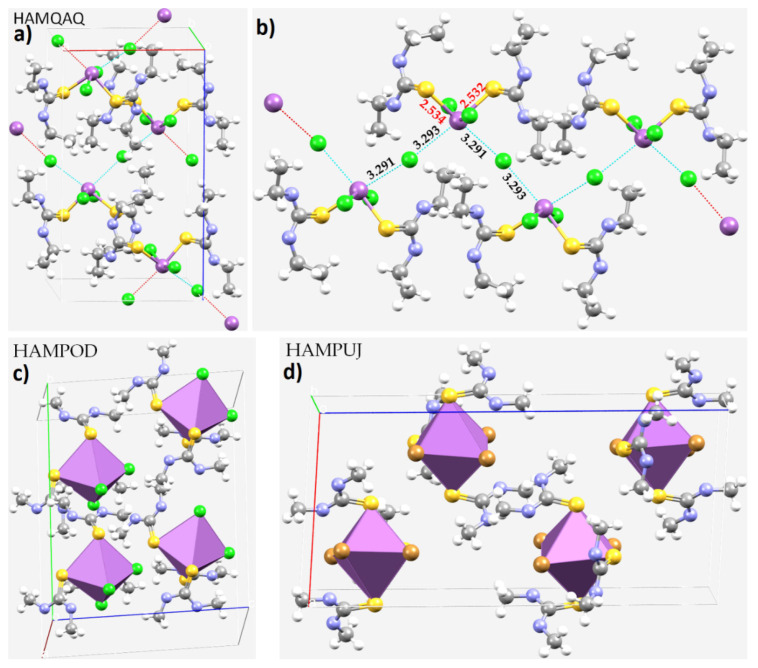
(**a**) The mixed polyhedral and ball-and-stick model of the unit-cell crystal structure of b,c,d-Cl-[SbCl_3_(DETU)_2_]. (**b**) The nature of Sb···Cl zigzag array responsible in part for the packing of the entire crystal that extends along the crystalgraphic *a*-axis. The polyhedral models of the crystal structure of (**c**) *fac*-SbCl_3_(DMTU)_3_ and (**d**) *mer*-SbBr_3_(DMTU)_3_]. Selected bond lengths and bond angles in Å and degree, respectively. The CSD ref. code is shown for each case. The polyhedra represent to a six-coordinate Sb, and atoms are shown as small spheres and bonds as lines in atom color, whereas the intermolecular interactions are represented by dotted lines.

Antimony(III) complexes (SbX_3_, X = Cl and Br) with N-substituted thioureas *N,N*-dimethylthiourea (DMTU) and *N,N*-diethylthiourea (DETU) have been reported [156]. They include *fac*-SbCl_3_(DMTU)_3_ and *mer*-SbBr_3_(DMTU)_3_], with octahedral Sb^3+^. In b,c,d-Cl-[SbCl_3_(DETU)_2_], the geometry of Sb^3+^ is square pyramidal, with two sulfur atoms from two DETU and three chlorides making up the coordination sphere. The sixth coordination site of Sb^3+^ is occupied by long *μ*–Cl···Sb interactions, leading to a polymeric structure. These Sb···Cl bond distances are 3.291 and 3.293 Å, i.e., substantially longer than the two Sb–Cl bonds (2.573 and 2.608 Å) and the two Sb–S bonds (2.532 and 2.534 Å). The unit-cell of the crystal is shown in Figure 24a, and the nature of the Sb···Cl zigzag array which is responsible in part for the packing of the entire crystal that extends along the crystalgraphic *a*-axis is shown in Figure 24b. We recognize these longer Sb···Cl bonds along the S–Sb bond extensions as pnictogen bonds; they are Type-IIa pseudo-linear interactions (∠S–Sb···Cl = 168.7° for the bond with *r*(Sb···Cl) = 3.293 Å and 175.3° for the bond with *r*(Sb···Cl) = 3.291 Å). 

In *fac*-SbCl_3_(DMTU)_3_ and *mer*-SbBr_3_(DMTU)_3_ (Figure 24c and Figure 24d, respectively), there are three Sb–S and three Sb–X (X = Cl, Br) bonds. There are also long Sb···X contacts (one *r*(Sb···Cl) = 3.011 Å in *fac*-SbCl_3_(DMTU)_3_ and two *r*(Sb···Br) = 3.006 and 3.180 Å contacts in *mer*-SbBr_3_(DMTU)_3_. 

### 8.12. Crown Ethers as Pnictogen Bond Acceptor Hosts for the Formation of Stibium Bonds 

Crown ethers have played an important role in coordination chemistry, and notably in transition metal host–guest chemistry [113,157,158,159,160,161,162]. Their complexation with p-block metal halides has been reported several times (for example [160,163,164,165,166,167]). 

**Figure 25:**
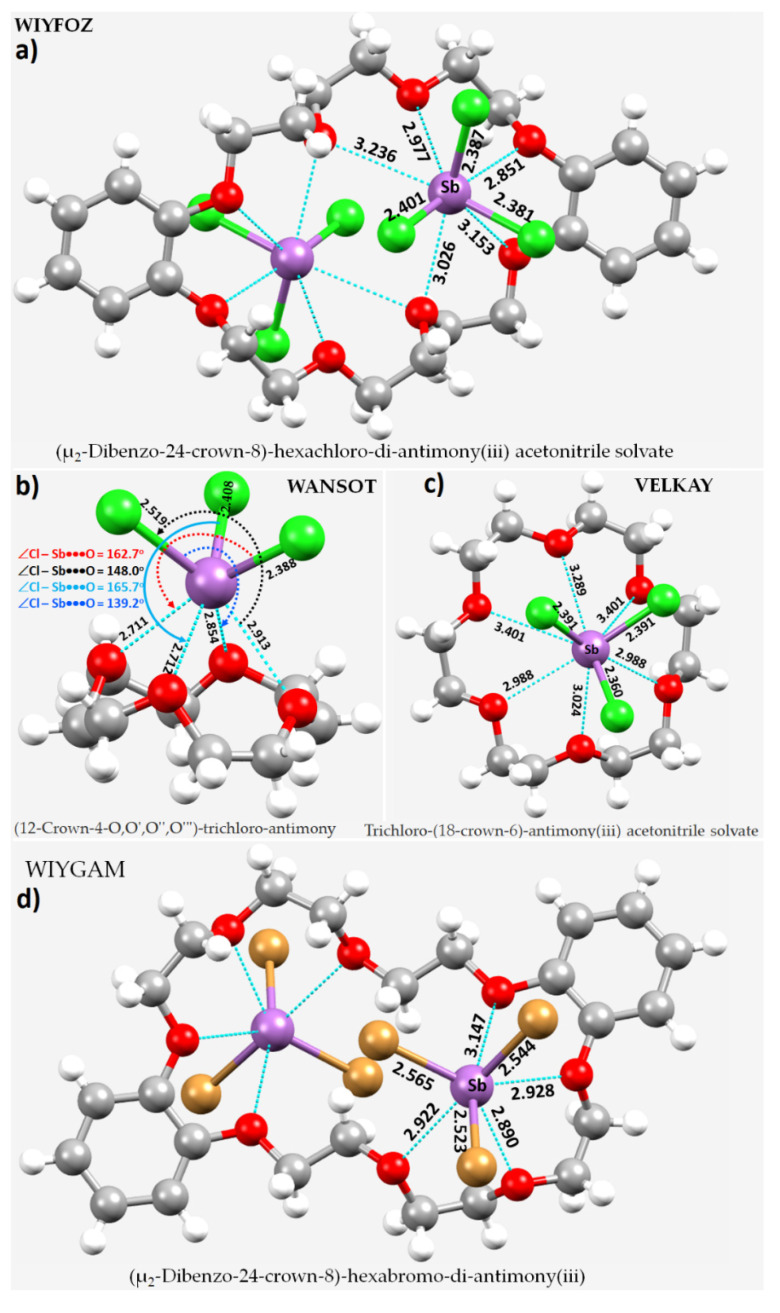
The local structure the crystals of (**a**) (dibenzo-24-crown-8)(SbCl_3_)_2_; [160] (**b**) (12-crown-4)SbCl_3_; [162] (**c**) (18-crown-6)SbCl_3_; [161] and (**d**) (dibenzo-24-crown-8)(SbBr_3_)_2_. [160] Selected bond distances and bond angles are shown in Å and degree, respectively. Solvent acetonitrile in (**a**) is omitted for clarity. The CSD ref. code in capital letters is shown for each case. Atoms are shown as small spheres and bonds as lines in atom color, whereas the intermolecular interactions are represented by dotted lines in cyan.

Shown in Figure 25 are the complexes of SbX_3_ (X = Cl, Br) with dibenzo-24-crown-8, 18-crown-6, and 12-crown-4. The large cavity of dibenzo-24-crown-8 shows the ability to accommodate two SbX_3_ units (Figure 25a,d). The O lone-pairs of the crown ether face the electrophilic regions on SbBr_3_ (see Figure 25d), leading to the formation of four Sb···O contacts of between 2.893(7) and 3.183(8) Å), while the hydrophobic methylene bridges face the three Br sites in the neighboring SbBr_3_ unit with Sb–Br bond lengths of 2.5268(15), 2.5478(15), and 2.5558(14) Å). The Sb···O contacts may not represent coordinate bonds, since the length the contacts (for example, the 3.147 Å contact) would suggest that these are antimony-centered pnictogen bonds. Two of them are quasi-linear, appearing along the Br–Sb bond extensions, while the other two are bent. The strength of these interactions may fall into the border region between coordinate and very strong pnictogen bonds. 

The bonding pattern in the dibenzo-24-crown-8 complex with SbCl_3_ is somewhat different (Figure 25a) relative to that found with SbBr_3_ (Figure 25d). There are now five Sb···O close contacts; no doubt, this is a consequence of the smaller size of SbCl_3_ compared to SbBr_3_. The three formal Sb–Cl bonds of SbCl_3_ have bond lengths of 2.3807(8), 2.3874(8), and 2.4015(9) Å) that are predominantly shorter. Because the electrostatic surface along the X–Sb bond extensions is positive (cf. Figure 2b,c), it links to the O-sites from opposite sides of the cavity of the crown-ether. This results in a sigmoidal conformation, in which two pseudo O_5_ donor cavities are created to satisfy the host–guest matching requirements. With this topology of bonding, the two O sites of the dibenzo-24-crown-8 serve as bifurcated pnictogen bond acceptors with Sb···O contacts in the range 2.852(2)–3.153(2) Å. Regardless of the nature of guest, i.e., SbCl_3_ or SbBr_3_, most of the Sb···O pnictogen bonds between SbX_3_ (X = Cl, Br) with dibenzo-24-crown-8 were found to be non-linear Type-II. 

**Figure 26:**
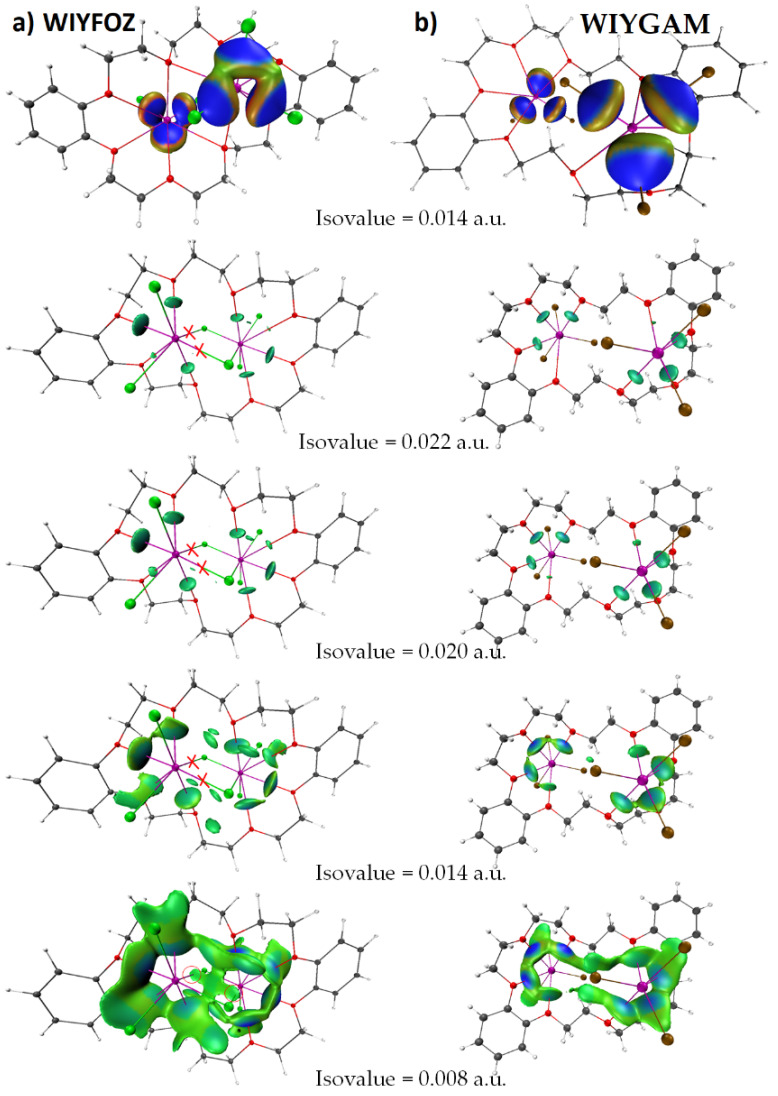
(**Top**) IGM-δ*g* based isosurface plots between Sb and X in SbX_3_: (**a**) (dibenzo-24-crown-8)(SbCl_3_)_2_ and (**b**) (dibenzo-24-crown-8)(SbBr_3_)_2_. (**Bottom**) IGM-δ*g* based isosurface plots between Sb and O in the corresponding systems, respectively, showing the dependence of the isosurfaces on the IGM isovalues. The crosses on the left plots indicate the disappearance of Sb···Cl bonds for high isovalues.

In order to provide further insight into the coordination chemistry of Sb^3+^ ions, we performed IGM-δ*g* analyses on their crystal geometries; our results are shown in Figure 26. The isosurface volumes in the plots shown in the top graphs were obtained between the Sb and X atoms in SbX_3_. They are colored deep blue, indicative of a reasonably high concentration of charge density in the bonding regions between Sb and X, as expected for coordinate bonds in SbX_3_. The remaining IGM-δ*g* based isosurface plots of Figure 26 correspond to different isovalues, illustrating that intermolecular interactions are not faint. That is, the isosurfaces for the very strong interactions between Sb and O always appear, regardless of the isovalue used. They are bluish-green in nature, and demonstrate the stability anticipated from the intermolecular bond distances. However, the isosurfaces corresponding to the longer bonds formed between Sb in one unit of SbCl_3_ and Cl in the second unit of the same molecule do not show up for large isovalues (0.022, 0.020 and 0.14 a.u.). This is not very surprising given that small isovalues are necessary to visualize the weak and van der Waals interactions that correspond to regions with a very low charge density gradient.

When an isovalue of 0.008 a.u. was used, most of the chemical interactions between SbCl_3_ and dibenzo-24-crown-8 were revealed. In addition to several H···Cl interactions, we observed two additional Sb···Cl interactions between the two SbCl_3_ units marked by circles in Figure 26a (bottom plots). They are longer (*r*(Sb···Cl) = 3.957 Å each) and bent (∠Cl–Sb···Sb(Cl) = 76.4°). These results suggest that antimony in (dibenzo-24-crown-8)(SbCl_3_)_2_ is eight-fold coordinated. At least three of the contacts (one Sb···Cl and two Sb···O_crown_) are of weak-to-medium strength and are stibium bonds.

A similar result was obtained with the IGM-δ*g* based isosurface analysis for (dibenzo-24-crown-8)(SbBr_3_)_2_, but there are no (Br–)Sb···Br links between the two SbBr_3_ units. However, the dependence of the size of the isosurface on the isovalue was notable (see Figure 26b, bottom ones); there were no IGM-δ*g* isosurfaces found between Sb and O for an isovalue of 0.022 a.u., and they were revealed only when an isovalue < 0.020 a.u. was used, suggesting that they are genuine medium-strength pnictogen bonds.

Figure 25b,c display the local bonding environment between the host and guest species in (12-crown-4)SbCl_3_ [162] and (18-crown-6)SbCl_3_ [161], respectively. Because the cavity of 12-crown-4 is smaller than that of 18-crown-6, the Sb^3+^ in SbCl_3_ forms four Sb···O contacts in its complex with the first, but six with the second, resulting in a bowl-shaped architecture with a cone-like facial stand formed by SbCl_3_. 

**Figure 27:**
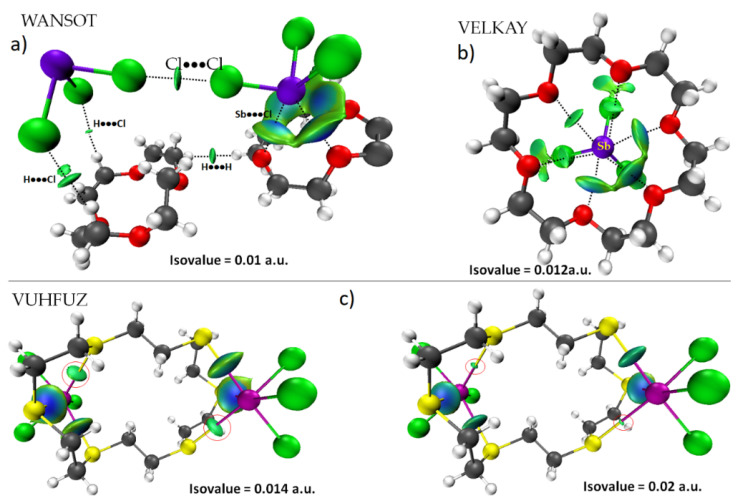
IGM-δ*g* based isosurface plots for (**a**) (12-crown-4)SbCl_3_, (**b**) (18-crown-6)SbCl_3_ and (**c**) (18-S-6)(SbCl_3_)_2_. The isosurface plots in (**c**) were obtained with two different isovalues.

As a means of verifying these tentative deductions concerning the nature of the bonding in these systems, the IGM-δ*g* isosurfaces were calculated and are shown in Figure 27. There are four thick, bluish circular volumes between Sb and O atoms in Figure 27a for (12-crown-4)SbCl_3_ that signify Sb–O covalent bonds [162]. In addition, we note interactions between neighboring (12-crown-4)SbCl_3_ units: a Cl···Cl halogen bond; H···Cl hydrogen bonds between the methylene hydrogens of the crown ether and the negative sites on Cl of SbCl_3_; and H···H dihydrogen type attractive interactions between the methylene groups of the crown ether (Figure 27a). In the case of (18-crown-6)SbCl_3_, there are three greenish and three bluish-green thick circular volumes between Sb and O (Figure 27b). The first three are pnictogen bonds and the others are genuine coordinate bonds. In addition, there are several H···Cl hydrogen bound engagements between SbCl_3_ and 18-crown-6. However, it should be kept in mind that these are not just the interactions responsible for the stability of the infinite crystals. There are a number other interactions between the host and guest species, including H…O hydrogen bonds, which can be revealed when the unit-cell is expanded. 

There are five Sb···O pnictogen bound contacts between the interacting units in the crystal structure of (cyclohexyl-15-crown-5)SbCl_3_ [114] (CSD ref. GESDOA), with *r*(Sb···O) varying between 2.825 and 3.002 Å (cf. Figure 1g). They are indeed longer than the three Sb–Cl coordinate bonds, i.e., 2.419, 2.427 and 2.432 Å. Of the five Sb···O pnictogen bonds, three are quasi-linear (∠Cl–Sb···O = 154.6°, 165.9° and 168.3°) and the remaining two are non-linear (∠Cl–Sb···O = 135.8° and 144.7°). Other than these, there are several H···Cl hydrogen bonding interactions between the –CH_2_ fragments of cyclohexyl-15-crown-5 and the Cl atoms of SbCl_3_.

In the complex between SbCl_3_ and 18-S-6 (18-S-6 = 1,4,7,10,13,16-hexathiacyclooctadecane), (18-S-6)(SbCl_3_)_2_, the large thio-crown accommodates two SbCl_3_ units, with three Sb···S contacts of 2.968, 3.061 and 3.461 Å, i.e., longer than the Sb–Cl coordinate bonds (2.471, 2.402 and 2.381 Å), and with ∠Cl–Sb···S = 141.7° [109]. These longer-range interactions are clearly pnictogen bonds. The IGM-δg based isosurface analysis shown in Figure 27c suggests that two of the Sb–S links are coordinate bonds and the remaining long Sb···S contact is a pnictogen bond. The isosurface for this contact becomes very faint when a high isovalue of 0.02 a.u. was used in our IGM-δ*g* analysis.

**Figure 28:**
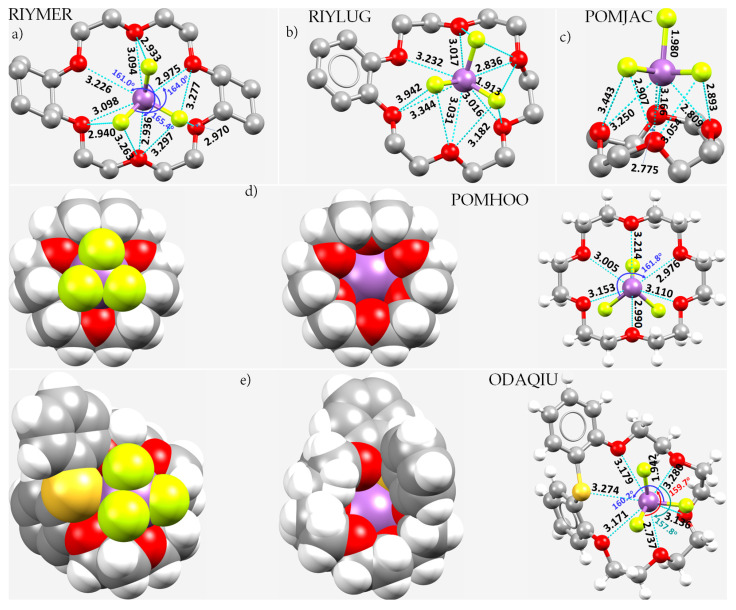
(**a**–**e**) Examples of some host–guest complexes of SbF_3_ with crown-ethers. Selected bond lengths and bond angles are in Å and degrees, respectively. (**a**) (*cis-anti-cis*-dicyclohexyl-8-crown-6)SbF_3_ [168]; (**b**) (benzyl-18-crown-6)SbF_3_ [168]; (**c**) (12-crown-4)SbF_3_ [169]; (**d**) (18-crown-6)SbF_3_ [169]; and (**e**) ([1.5]dibenzothia-18-crown-6)SbF_3_ [170]. For clarity, the H-atoms are omitted in the first two cases, and potential secondary interactions are omitted for most cases. Atom type is shown in some cases, and the CSD ref. code is given. Benzene solvate is not included in (**e**) for clarity. Selected bong lengths and bond distances are shown in Å and degree, respectively.

On the other hand, the intermolecular bonding modes of crown-ethers with SbF_3_ are somewhat different to those of the other antimony trihalides, no doubt because of the small, highly electronegative nature of fluorine. Host–guest fitting is more prominent for SbF_3_ with the bound F atoms of the molecule involve in additional Sb–F···O interactions when a large crown-ether is the host (Figure 28a–e). These interactions are either comparable to, or slightly longer than, the F–Sb···O interactions, but are markedly longer than the Sb–F bonds (*r*(Sb–F) ~ 1.9 Å). The former are Type-I and the latter are Type-IIa interactions, as deduced from our investigation of the ∠Sb–F···O and ∠F–Sb···O contact angles in the solid-state structures of SbF_3_ complex with a number of 18-crown-6 crown ethers (Figure 28d,e). 

The ∠F–Sb···O angles formed along the three F–Sb bond extensions are quasi-linear, and within the ranges 161–166°, 159.2–167.5°, and 161–165.0° in the complexes (*cis-anti-cis*-dicyclohexyl-8-crown-6)SbF_3_ [168], (benzyl-18-crown-6)SbF_3_ [168], and (12-crown-4)SbF_3_ [169], respectively (see Figure 28a, Figure 28b and Figure 28c, respectively). The remaining F–Sb···O interactions are non-linear. For example, ∠F–Sb···O lies between 130° and 133° for three F–Sb…O bonds that are significantly off the F–Sb axes in (*cis-anti-cis*-dicyclohexyl-8-crown-6)SbF_3_ [168], and between 161–165.0° along the remaining three F–Sb···O bonds in (18-crown-6)SbF_3_ [169]. The values of ∠F–F···O are in the range 60° and 90° for all these complexes. 

The SbF_3_ molecule does not fit well inside the cage provided by (12-crown-4)SbF_3_; see Figure 28c [169]. Consequently, the ∠F–Sb···O contact angles deviate significantly from linearity. For instance, the four (F–)Sb···O intermolecular distances of 2.775, 2.809, 3.166, and 3.250 Å correspond ∠F–Sb···O contact angles of 151.7°, 133.7°, 123.1°, and 137.4°, respectively. We, therefore, refer to these F–Sb···O interactions in this and similar other systems as “non-linear pnictogen bonds”. Most of the Sb–F···O interactions in (12-crown-4)SbF_3_ are also non-linear, except for one interaction which is highly directional (r(F···O)) = 2.907 Å; ∠Sb–F···O = 66.3°; ∠C–O···F = 158.8°), and the directional nature of the interaction is determined based on the C–O···F angle. 

We note further that in all these host–guest structures, the importance of secondary interactions cannot be overlooked. They appear either as H···X or F···O interactions between SbX_3_ and crown-ether. The presence of H···X hydrogen bonds in the host–guest complex systems can be inferred from the space-filling models shown in Figure 28d; each covalently-bound F in SbF_3_ kisses at least two nearest H atoms of the -CH_2_ fragment of 18-crown-6, forming a set of six hydrogen bonds. Of course, the number and extent of the formation of hydrogen bonds is determined by the size of the crown-ether. That SbF_3_ molecular entity fits well inside the 18-crown-6 cavity is also evident in Figure 28d (see the space filling model, extreme left). 

**Figure 29:**
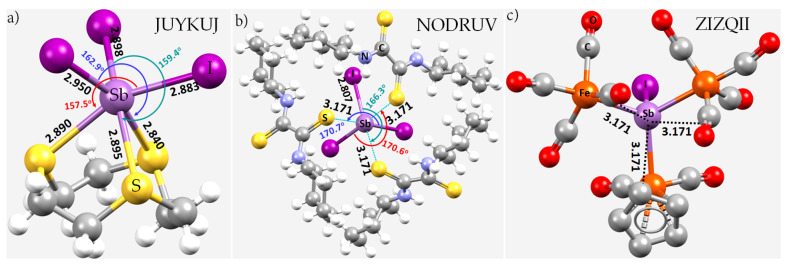
Examples of some host–guest complexes of (**a**) (HDTOA)_3_(SbI_3_)_2_ (HDTOA = *N,N’*-dicyclohexyldithio-oxamide) [171]; (**b**) (9-S-3)SbI_3_ (9-S-3 = 1,4,7-trithiacyclononane) [172]; (**c**) [(η^5^-cyclopentadienyl-Fe(CO)_2_)(Fe(CO)_4_)_2_SbI]^−^ [173]. For clarity, potential secondary interactions are omitted in most cases. Atom type is shown in some cases, and the CSD ref. code is given. Selected bond lengths and distances are shown in Å and degree, respectively.

In the case of the crystal structure of (HDTOA)_3_(SbI_3_)_2_ (HDTOA = *N,N’*-dicyclohexyldithiooxamide) [171] (Figure 29a), the three Sb–I bonds of SbI_3_ are about 0.36 Å shorter than the remaining three Sb···S contacts. The pseudo-octahedral coordination sphere of Sb^3+^ is completed by interaction with three S donors from three different HDTOA ligands in quasi-linear contacts (∠I–Sb···S = 166–171°), characteristic of pnictogen bonds. 

The intermolecular interaction between 9-S-3 crown ether and SbI_3_ in the 1:1 adduct (9-S-3)SbI_3_ (9-S-3 = 1,4,7-trithiacyclononane) (Figure 29b) is comparatively stronger, as attested to by the shorter (I–)Sb···S bond distances. The Sb^3+^ lone pair is not stereochemically active [172]. Indeed, the Sb···S distances are shorter than the Sb–I bond lengths. This suggests that the Sb···S contacts may be regarded as very strong pnictogen bonds.

By contrast, in the crowded environment of (NEt_4_) [(η^5^-cyclopentadienyl-Fe(CO)_2_)(Fe(CO)_4_)_2_SbI] [173], tetra-coordinated Sb^3+^ also participates in Sb···π(C≡O) interactions (Figure 29c). There are six such interactions, with each pair developing along the non-linear extension of each Sb–X (X = I, Fe) bond. The Sb···π(mid of point of C≡O) distance is 3.399 Å, and Sb···C(≡O) distance is 3.171 Å. From an examination of the space-filling model of the system, it was inferred that the Sb cation was also linked non-covalently with the near C sites of the Fe-bound η^5^-cyclopentadienyl moiety. 

**Figure 30:**
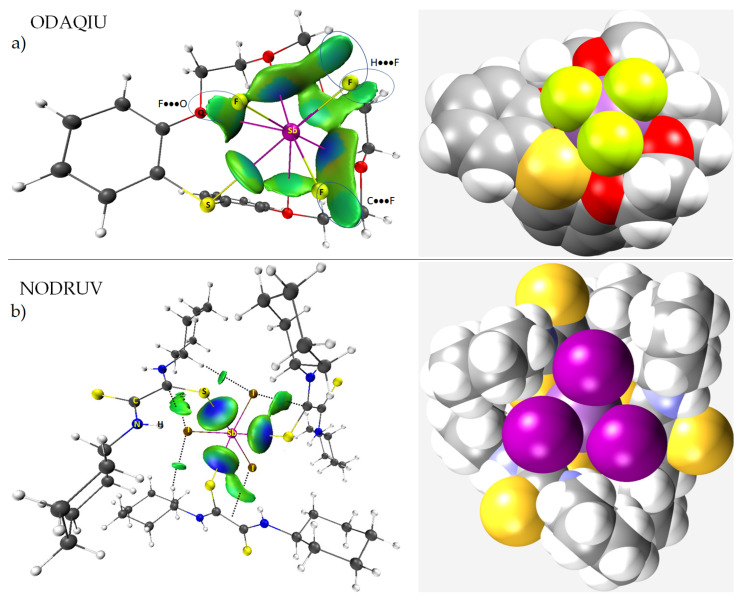
IGM-δ*g* based isosurface plots for (**a**) [1.5]dibenzothia-18-crown-6)SbF_3_ [170], and (**b**) (HDTOA)_3_(SbI_3_)_2_ (HDTOA = *N,N’*-dicyclohexyldithio-oxamide) [171]. Atom labeling is shown. Isosurfaces colored blue and green between the interacting molecules in the crystal signify strong and weak intermolecular interactions, respectively. Other intermolecular interactions (viz. hydrogen bonds, C···F, F···O and C···I bonds, etc.) are marked with dotted lines in (**b**) and circles in (**a**). The space-filling model is shown for each case.

To verify these conclusions, we examined the IGM-δ*g* isosurfaces. Two examples are shown in Figure 30. The top one, Figure 30a, is for [1.5]dibenzothia-18-crown-6)SbF_3_ [170], while the bottom one, Figure 30b, is for (HDTOA)_3_(SbI_3_)_2_ (HDTOA = *N,N’*-dicyclohexyldithio-oxamide) [171]. The IGM-δ*g* based isosurface results are in agreement with the inferences drawn from the intermolecular bond distances between the interacting molecules (Figure 29a,b) and the MESP analysis of SbI_3_ (see Figure 2d). In the case of [1.5]dibenzothia-18-crown-6)SbF_3_, of the six Sb···O contacts, the two with the shortest distance are genuine coordinate bonds, as evidenced by the bluish-green isosurfaces. The other four Sb···O contacts are associated with greenish isosurfaces and are seemingly pnictogen bonds. In addition, we observed F···O, C···F and H···F interactions between SbF_3_ and the crown ether. All the Sb–I and Sb–S contacts in (HDTOA)_3_(SbI_3_)_2_ are coordinate bonds, since they feature bluish-green isosurfaces. We have also observed a number of (N)C···I/(C)C···I and H···I contacts between the interacting monomer units. These results are in agreement with what might have been speculated from the space-filling models that rely on the vdW radii of the atomic domains. 

## 9. Conclusions

In this overview, we have examined the nature of the electrostatic surfaces of several molecular entities in various illustrative crystal lattices to provide evidence of the occurrence of an often overlooked non-covalent interaction, the antimony-centered pnictogen bond, or more succinctly, the stibium bond. We must emphasize that we do not claim that our survey of the CSD and ICSD databases is comprehensive; we have merely drawn illustrative examples from those databases to highlight the occurrence of overlooked non-covalent interactions.

Pnictogen bonding in crystal lattices has been known since the middle of the last century, so it is misleading to claim that it only became known to community after 2011.Our investigations of intermolecular distances, directionality, electrostatic potential and IGM-δ*g* based isodensity surfaces in most of the selected systems have enabled to us demonstrate that hypervalent Sb atoms in molecular entities contain more than one *σ*-hole and have the potential for involvement in attractive engagements with the negative sites in neighboring molecular entities, thereby causing (or contributing to) the assembly and stability of crystal lattices. The MESP model is suitable for understanding of directional Type-II interactions that are Coulombic, but not for rationalizing Type-I or -III interactions; this may be overcome by applying the IGM-δ*g* model. The “sum of the vdW radii” concept, together with other geometric and topological features, has shown to be very effective in detecting pnictogen bonding in crystals when applied carefully.

Depending on the molecular environment, the directionality of these interactions may appear in different flavors, following Type-I, -II and -III bonding topology interactions. Our investigation suggests that pnictogen bonds are not always linear, or quasi-linear; indeed, non-linear Type-II interactions were abundant in many of the crystals we examined. In most cases, other primary or secondary interactions (such as hydrogen bonding, halogen bonding, tetrel bonding and other π-centered interactions) occur simultaneously, which drive the directionality of the stibium bonds. The involvement of the same covalently bound Sb atom in multiple interactions (for example, as in crown-ether complexes) and the competition between them influence the directionality of the pnictogen bonds. 

We have demonstrated that antimony in molecules can form pnictogen bonds when it finds itself in the close vicinity of Lewis bases such as O, N, F, Cl, Br, I and S in molecules with which it interacts. We have also shown that pnictogen bonds can be formed with the delocalized π-density on the fragments –C=N=S and –C≡O in molecular entities, as well as on arene moieties. These findings suggest that donors of pnictogen bonds are no different to those that have been widely examined for hydrogen bonds, halogen bonds, and other non-covalent interactions. None of the crystal systems showed evidence that stibium bonds were the only attractive interactions causing molecular assembly and therefore contributing to the entirety of crystal stability.

We have also observed that intramolecular stibium bonds in crystals are not so uncommon, even though intermolecular stibium bonds are widely present in a variety of crystals. While the majority of the illustrative systems presented in this overview have yet to be thoroughly explored using state of the art computational methods and other approaches, we expect that this work will serve as a guide for researchers who use, or intend to use, pnictogen bonding in their work. Since Sb has already played a significant role in the synthetic design of functional zero-, one-, two- and three-dimensional nano-scale materials, especially in the area of optoelectronics, the possibility of exploiting the Sb-centered pnictogen bonding interactions discussed in this study should assist future investigations into these and other materials.

## Figures and Tables

**Table 1:** The van der Waals radius (Å) of some selected atoms proposed by different authors.

Atom Number Z	Atom Symbol	Bondi [69]	Batsanov [70]	Alvarez [68]	Mantina et al. [71]
1	H	1.20	---	1.20	1.10
6	C	1.70	1.70	1.77	1.70
7	N	1.55	1.60	1.66	1.55
8	O	1.52	1.55	1.50	1.52
9	F	1.47	1.50	1.46	1.47
15	P	1.80	1.95	1.90	1.80
16	S	1.80	1.80	1.89	1.80
17	Cl	1.75	1.80	1.82	1.75
33	As	1.85	2.05	1.88	1.85
35	Br	1.83	1.90	1.86	1.83
51	Sb	---	2.20	2.47	2.06
53	I	1.98	2.10	2.04	1.98
83	Bi	---	2.30	2.54	2.07

**Table 2:** Comparison of MP2(full)/def2-TZVPPD level 0.001 a.u. isodensity envelope mapped potentials with those of ωB97XD/def2-TZVPPD potentials computed on the surfaces of the SbX_3_ molecules (X = F, Cl, Br, I).

Local Most Extrema on the Surface of Specific Atom/Bond	SbF_3_		SbCl_3_		SbBr_3_		SbI_3_	
	MP2(Full)	ωB97XD	MP2(Full)	ωB97XD	MP2(Full)	ωB97XD	MP2(Full)	ωB97XD
*V_s,min_* on X (lateral portions)	−22.1	−21.9	−10.3	−10.5	−8.8	−9.0	−6.9	−7.1
*V_s,min_* on Sb (opposite to the triangular face formed by three X atoms)	31.2	30.7	25.0	24.9	23.1	22.9	20.0	19.2
*V_S,max_* (on Sb–X bond extensions)	---	---	1.6	1.8	6.5	7.2	12.3	14.3
*V_S,max_* (on X–Sb bond extensions)	48.2	48.6	38.4	39.7	34.7	36.3	29.3	31.1
*V_S,max_* (on the centroid of the triangular face formed by three X atoms)	3.6	5.7	0.5	2.4	0.3	2.4	0.5	2.5

## Data Availability

This research did not report any data.
